# A novel signature of cuproptosis-related lncRNAs predicts prognosis in glioma: Evidence from bioinformatic analysis and experiments

**DOI:** 10.3389/fphar.2023.1158723

**Published:** 2023-04-10

**Authors:** Di Chen, Yuan Xu, Xueping Gao, Xuqiang Zhu, Xianzhi Liu, Dongming Yan

**Affiliations:** ^1^ Department of Neurosurgery, The First Affiliated Hospital of Zhengzhou University, Zhengzhou, Henan, China; ^2^ School of Basic Medicine, Gannan Medical University, Ganzhou, Jiangxi, China; ^3^ Department of Clinical Laboratory Medicine, Southwest Hospital, Third Military Medical University (Army Medical University), Chongqing, China

**Keywords:** cuproptosis, long non-coding RNA, prognostic signature, glioma, immune infiltration, therapeutic response prediction, LEF1-AS1

## Abstract

**Background:** Glioma patients often experience unfavorable outcomes and elevated mortality rates. Our study established a prognostic signature utilizing cuproptosis-associated long non-coding RNAs (CRLs) and identified novel prognostic biomarkers and therapeutic targets for glioma.

**Methods:** The expression profiles and related data of glioma patients were obtained from The Cancer Genome Atlas, an accessible online database. We then constructed a prognostic signature using CRLs and evaluated the prognosis of glioma patients by means of Kaplan-Meier survival curves and receiver operating characteristic curves. A nomogram based on clinical features was employed to predict the individual survival probability of glioma patients. Functional enrichment analysis was conducted to identify crucial CRL-related enriched biological pathways. The role of LEF1-AS1 in glioma was validated in two glioma cell lines (T98 and U251).

**Results:** We developed and validated a prognostic model for glioma with 9 CRLs. Patients with low-risk had a considerably longer overall survival (OS). The prognostic CRL signature may serve independently as an indicator of prognosis for glioma patients. In addition, functional enrichment analysis revealed significant enrichment of multiple immunological pathways. Notable differences were observed between the two risk groups in terms of immune cell infiltration, function, and immune checkpoints. We further identified four drugs based on their different IC50 values from the two risk groups. Subsequently, we discovered two molecular subtypes of glioma (cluster one and cluster two), with the cluster one subtype exhibiting a remarkably longer OS compared to the cluster two subtype. Finally, we observed that inhibition of LEF1-AS1 curbed the proliferation, migration, and invasion of glioma cells.

**Conclusion:** The CRL signatures were confirmed as a reliable prognostic and therapy response indicator for glioma patients. Inhibition of LEF1-AS1 effectively suppressed the growth, migration, and invasion of gliomas; therefore, LEF1-AS1 presents itself as a promising prognostic biomarker and potential therapeutic target for glioma.

## 1 Introduction

It has been estimated that glioma are the foremost cause of fatality among individuals with brain neoplasms (Weller et al., 2015). Currently, the treatment options for numerous glioma patients involve maximal safe surgical resection, concomitant with postoperative radiotherapy and chemotherapy (Ostrom et al., 2014). Nonetheless, the outlook for glioma patients is far from satisfactory, especially for those with glioblastoma (GBM), where the 5-year relative survival rate is a mere 5% (Ostrom et al., 2014). Additionally, the low-grade glioma (LGG) has a high likelihood of transforming into high-grade gliomas, further underscores the unsatisfactory prognosis for glioma (Louis et al., 2016). This unfavorable prognosis is primarily due to the infiltrative nature of gliomas, acquired resistance, high recurrence rates, and intratumoral heterogeneity (Hou et al., 2006; Rajesh et al., 2017). The rapid development of molecular biological technology has not only enhanced our comprehension of glioma pathogenesis, but also revealed genetic alterations and critical targetable pathways. To date, there has been substantial interest in studying molecular therapeutic strategies that may provide renewed hope and perspectives for glioma patients (Rajesh et al., 2017). Accordingly, identifying new prognostic factors and targets for gliomas is imperative.

It has been reported that pyroptosis (Chen et al., 2021), necroptosis (Liu et al., 2022a; Yuan et al., 2022) and ferroptosis (Wan et al., 2021) are pivotal in the development of glioma. For instance, genes associated with pyroptosis have been demonstrated to serve as prognostic indicators and signify the molecular characteristics of distinct subtypes of glioma (Chen et al., 2021). Further researches have indicated that genes related to necroptosis can act as predictive markers for glioma patients, resulting in an improved prognostic precision (Liu et al., 2022a; Yuan et al., 2022). Additionally, a novel risk score connected with ferroptosis-related genes has been reported to possess the capability of predicting the prognosis and immunotherapy outcomes of patients with glioma (Wan et al., 2021). Recently, a study has described cuproptosis (Tsvetkov et al., 2022), an alternative form of cell death in comparison to pyroptosis, necroptosis and ferroptosis (Chen et al., 2021; Wan et al., 2021; Liu et al., 2022a; Yuan et al., 2022). Copper can regulate intracellular homeostasis as a co-factor for multiple enzymes, but excessive copper accumulation may prove harmful and ultimately result in cell death (Kim et al., 2008). Previous study has established a strong correlation between copper accumulation and tumor cell growth, metastasis, and angiogenesis, hinting that cuproptosis could participate in the development of cancer (Brady et al., 2014; Blockhuys and Wittung-Stafshede, 2017). Chen et al. (2022) have developed an active cuproptosis score to determine the prognosis of glioma. Nevertheless, new biomarkers linked to cuproptosis for the prognosis and treatment of glioma have not yet been identified. Therefore, we are committed to identifying new biomarkers for the development of targeted therapies for glioma patients *via* this new modality of cell death.

Several investigations have established the association between long non-coding RNAs (lncRNAs) and the process of tumorigenesis (Qin et al., 2021). Furthermore, lncRNAs have demonstrated their utility as prognostic markers and potential therapeutic targets for glioma in various studies (He et al., 2021; Ba et al., 2022). For instance, researchers have identified and validated a lncRNA model of necroptosis that effectively predicts the survival of patients with glioma (Wu et al., 2022). Numerous studies have been published highlighting the ability of cuproptosis-related lncRNAs (CRLs) to predict the survival of patients with breast cancer (Jiang et al., 2022), hepatocellular carcinomas (Zhu et al., 2022) and renal clear cell carcinomas (Xin et al., 2022). However, to the best of our knowledge, there has been no systematic evaluation of whether CRLs can be utilized as biomarkers for the prognosis and treatment of glioma.

In this study, a clinical prognostic model consisting of 9 CRLs was generated and validated using bioinformatics. We subsequently evaluated the impact of the CRL signature on diverse cancer-related pathways and the tumor immune microenvironment. Finally, we identified LEF1-AS1 from the CRLs and tested the function of LEF1-AS1 in glioma. Therefore, LEF1-AS1 could serve as a significant prognostic biomarker and targeted treatment option for glioma.

## 2 Materials and methods

### 2.1 Data collection and identification of the prognostic CRLs

Transcriptomes and data of glioma patients including LGG and GBM were retrieved from the TCGA (https://portal.gdc.cancer.gov/) and CGGA (http://www.cgga.org.cn/) database. Eventually, 701 glioma samples from TCGA and 1,018 samples from CGGA (325 from the CGGA_325 cohort and 693 from the CGGA_693 cohort) were used as the study cohort (last accessed: 11 November 2022). A total of 19 cuproptosis-associated genes (CAGs) were identified based on previous publications (Tsvetkov et al., 2022) (Supplementary Table S1), and their expression and role in glioma were subsequently explored (Zhang et al., 2022a). Then, Pearson correlation analysis was used to evaluate the relationship between the expression levels of the 19 cuproptosis-related genes and those of lncRNAs (|Pearson correlation coefficient| > 0.4 and *p* < 0.001). We screened lncRNAs associated with overall survival (OS) in patients with glioma using univariate Cox regression analysis for prognostic identification subsequently.

### 2.2 Prognostic model construction and verification

The 701 glioma cases were randomized into a training set and a testing set in a 1:1 ratio of 1:1 for systematic analysis by the R package “caret” (Chen et al., 2022). The training set was utilized to build the CRL signature. Both the testing set and the entire set were used to validate the signatures. Moreover, the least absolute selection operator (LASSO) Cox regression algorithm analysis (using the penalty parameter estimated by 10-fold cross-validation and a *p*-value of 0.05) was performed by the R package “glmnet”. The risk score = Ʃ [Exp (lncRNA) x coef (lncRNA)]. Exp (lncRNA) and coef (lncRNA) present the corresponding expression level of each selected lncRNA and the regression coefficient, respectively. Based on the risk scores (with the median risk score used as a cutoff), all the glioma samples were separated into the low- and high-risk group. The prognosis of patients with gliomas was assessed by K–M curves and ROC curves (Sun et al., 2021).

### 2.3 Establishment of a nomogram for patients with glioma

Based on the independent prognostic factors (risk, age, and grade) in the TCGA cohort, we developed a nomogram to predict the patient’s OS (1-, 3-, and 5-year) by utilizing the R package “survival” and “regplot”. We also estimated the predictive power of the nomogram models using the consistency index (C-index) and calibration curve.

### 2.4 Analyses of PCA and GO, as well as KEGG

To visualize the spatial distribution of low- and high-risk samples, the gene expression profile, CAGs, CRLs, and risk model gene expression matrix were reduced in dimensionality through principal component analysis (PCA). Simultaneously, a 3D plot was generated with the R package “scatterplot3d” in our study to visualize the results. Then, we screened for differentially expressed genes (DEGs) by the R package “limma”, which filters for |log2fold-change (FC)| > 1 and adjusted *p* < 0.05. Moreover, we analyzed this DEGs using the “clusterProfiler” R package for Gene Ontology (GO) and Kyoto Encyclopedia of Genes and Genomes (KEGG) pathways (Huang et al., 2022).

### 2.5 Immune landscape analysis

The model and immune infiltration status were then compared by calculating the immune infiltration profiles (Liu et al., 2022b). We uncovered differences in the immune response using a heatmap under different algorithms. We also used single-sample GSEA (ssGSEA) to detect distinctions in immune cells infiltrating tumors and immune function between two groups of patients (Liu et al., 2022c). Furthermore, the immune checkpoint expression was examined in these two groups and shown in box plots. The stromal scores, ESTIMATE scores, and immune scores for each sample were calculated using the ESTIMATE algorithm *via* the R package “estimate” (Chen et al., 2018).

### 2.6 TMB, TIDE and evaluation of the therapeutic drug efficacy for the treatment of glioma

We determined the distinctions for the two groups concerning tumor mutation burden (TMB) by the R package “maftools” (Ba et al., 2022). Furthermore, our team estimated the difference in sensitivity to immunotherapy between patients in those two groups using the Tumor Immune Dysfunction and Exclusion (TIDE) scoring file obtained from the TIDE website (http://tide.dfci.harvard.edu/) (Jiang et al., 2018). Moreover, we used the half-maximal inhibitory concentration (IC50) to predict the sensitivity of gliomas to a common set of drugs using R packages that included “pRRophetic,” “limma,” “ggpubr,” and “ggplot2” with *p* = 0.001 (Geeleher et al., 2014). Correlation analysis was also conducted between each drugs and the risk score using Spearman correlation analysis.

### 2.7 Consensus clustering analysis

Consensus cluster analysis was implemented to elucidate the features of potential molecular subgroups responsive to immunotherapy according to prognosis-related CRL expression (Wilkerson and Hayes, 2010). The cumulative distribution function (CDF) approach was used to identify the molecular subtypes based on these prognostic CRLs. In the subsequent analysis, we examined the distinctions in survival probability and functions related to immunity among the two clusters.

### 2.8 Cell culture and reverse transcription quantitative PCR (RT-qPCR)

We acquired the normal control cell line (normal human astrocytes, NHA) and glioma cell lines (U251 and T98) from the National Collection of Authenticated Cell Cultures (Shang Hai, China) (Zhang et al., 2018). A TRIzol reagent was used to extract total cellular RNA (Invitrogen, Carlsbad, CA, United States). The data were normalized to glyceraldehyde-3-phosphate dehydrogenase (GAPDH) mRNA expression and calculated using the 2^−ΔΔCT^ method (Zhang et al., 2022b). The sequences of PCR primers are presented in Supplementary Table S2.

### 2.9 Cell transfection

A negative control (si-NC) and two LEF1-AS1-targeted siRNAs (si-LEF1-AS1) were generated by GenePharma (Shanghai, China). Six well plates were seeded with three hundred thousand U251 or T98 cells. The cells were transfected using Lipofectamine^®^ 3,000 (Invitrogen) after 24 h of incubation. Briefly, 2 μL of 100 μM siRNA, 5 μL of lipofectamine 3,000, 5 μL of P3,000, and 250 µL of Opti-MEM (Gibco) were mixed, incubated for 15 min, and then added to the 6-well plates. Each plate contained two positive tests (si-LEF1-AS1) and negative controls (si-NC) for each cell line. After incubation for 24 h, the cells were assayed for knockdown efficiency using qPCR. The siRNA sequences are presented in Supplementary Table S3.

### 2.10 Cell counting kit-8 assay and transwell assay

Cell counting kit-8 assay and transwell assay were performed as previously reported (Zhang et al., 2018). The cells well were cultured for 2 hours at 0, 1, 2, and 3 days in a 10 μLcell counting kit-8 (CCK-8) solution (Dojindo, Tokyo, Japan). A chamber of 24-well transwell plates was seeded with 300,000 transfected cells or control cells in DMEM without FBS to measure cell migration. To conduct the invasion experiment, precoating with Matrigel solution in a chamber was required. Finally, a microscope Olympus iX71 was used for counting and imaging the cells.

### 2.11 Statistical analysis

We used R software (version 4.1.3) to generate all statistical analyses and results. A chi-square test was performed for the classification variables between the training and testing sets. Pearson correlation was adopted to uncover the connections between clinical factors, risk scores, immune infiltration levels.

## 3 Results

### 3.1 Identification of prognostic CRLs in patients of glioma

**FIGURE 1:**
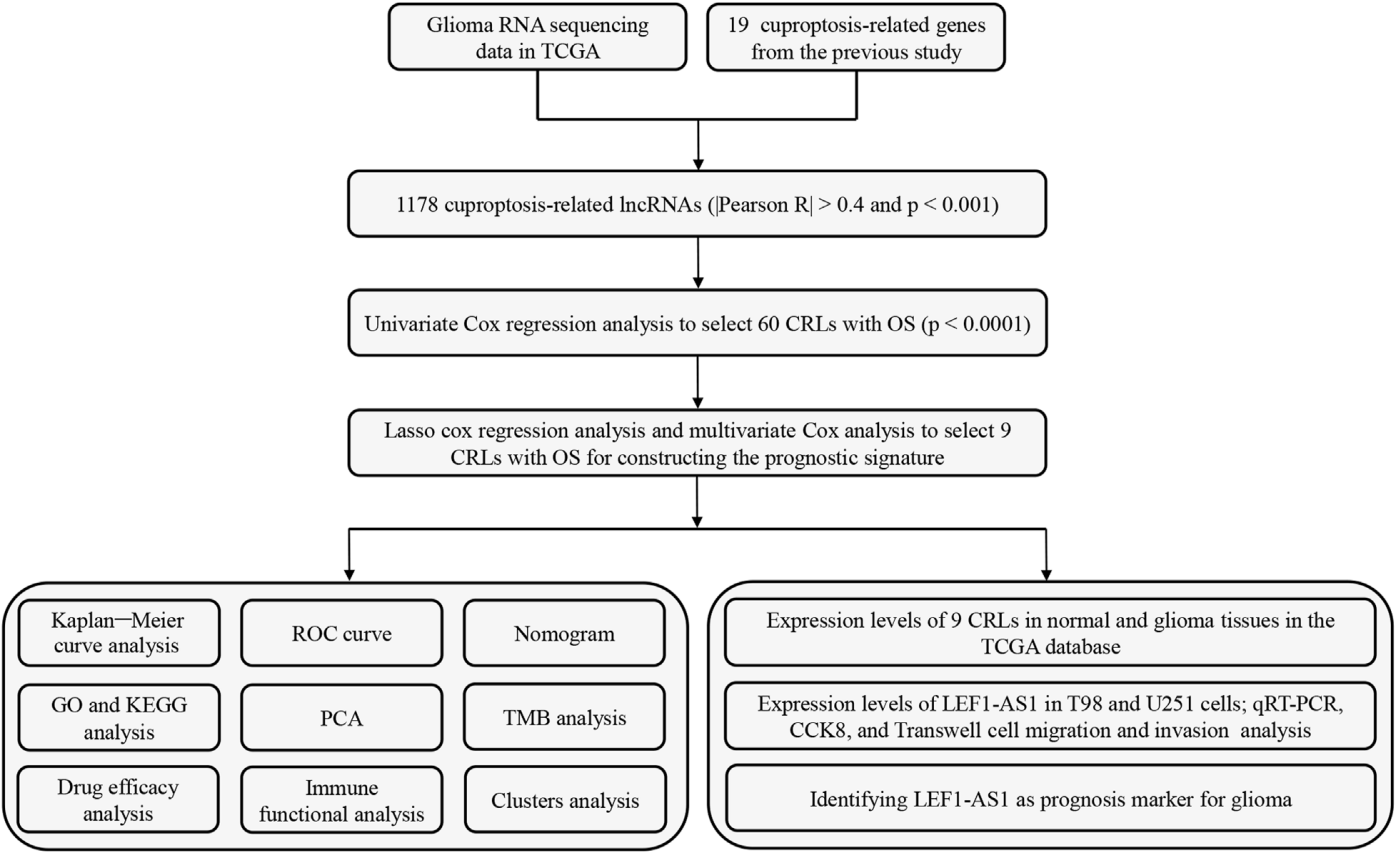
Flow diagram of the study design.

Figure 1 shows the flow diagram for the research. First, we screened 16,876 lncRNAs from the TCGA glioma database. There were 701 gliomas and five normal samples in the dataset. Using Pearson correlation analysis (Pearson R > 0.4 and *p* < 0.001), 1,178 CRLs showed large correlation with 19 CAGs. An interaction between CAGs and CRLs was visualized using Sankey diagrams (Supplementary Figure S1A). Finally, we identified 308 differentially expressed CRLs with log2 FC > 2 and *p* < 0.005. We generated a heatmap to visualize how CRLs are expressed differently in tumor and normal tissue (Supplementary Figure S1B).

### 3.2 Establishment and confirmation of the prognostic CRLs

The univariate analysis revealed the positive correlation between 60 CRLs and OS. As shown by the forest map (Supplementary Figure S1C) and heatmap (Supplementary Figure S1D), 19 CRLs were considered as factors related to poor prognosis for patients with glioma (hazard ratio [HR] > 1), whereas the remaining CRLs showed no association with increased risk. However, the Sankey diagram reveals a global trend for CRL upregulation (Supplementary Figure S2A). To decrease the risk of overfitting, we conducted a LASSO regression analysis on the CRLs (Supplementary Figures S2C, D). Furthermore, multivariate Cox regression analysis narrowed the count to 9 CRLs (HCG15, AC007950.2, PTPRN2-AS1, TRHDE-AS1, LEF1-AS1, AC021739.2, AC008915.2, ARHGAP42-AS1, and LINC01571) that were used to establish the OS prognostic signature. The Risk scores = (0.356486617130629 × HCG15 expression)− (0.733438839952268 × AC007950.2 expression) + (0.332295121384822 × PTPRN2-AS1 expression)− (0.910093784674468 × TRHDE-AS1 expression) + (0.499808915964012 × LEF1-AS1 expression)−(0.294440386492078 × AC021739.2 expression) + (0.455982744814798 × AC008915.2 expression)−(0.537789146806874 × ARHGAP42-AS1 expression) + (0.451090068728355 × LINC01571 expression). The association between CAGs and the 9 lncRNAs is also illustrated by the correlation heatmap (Supplementary Figure S2B).

**FIGURE 2:**
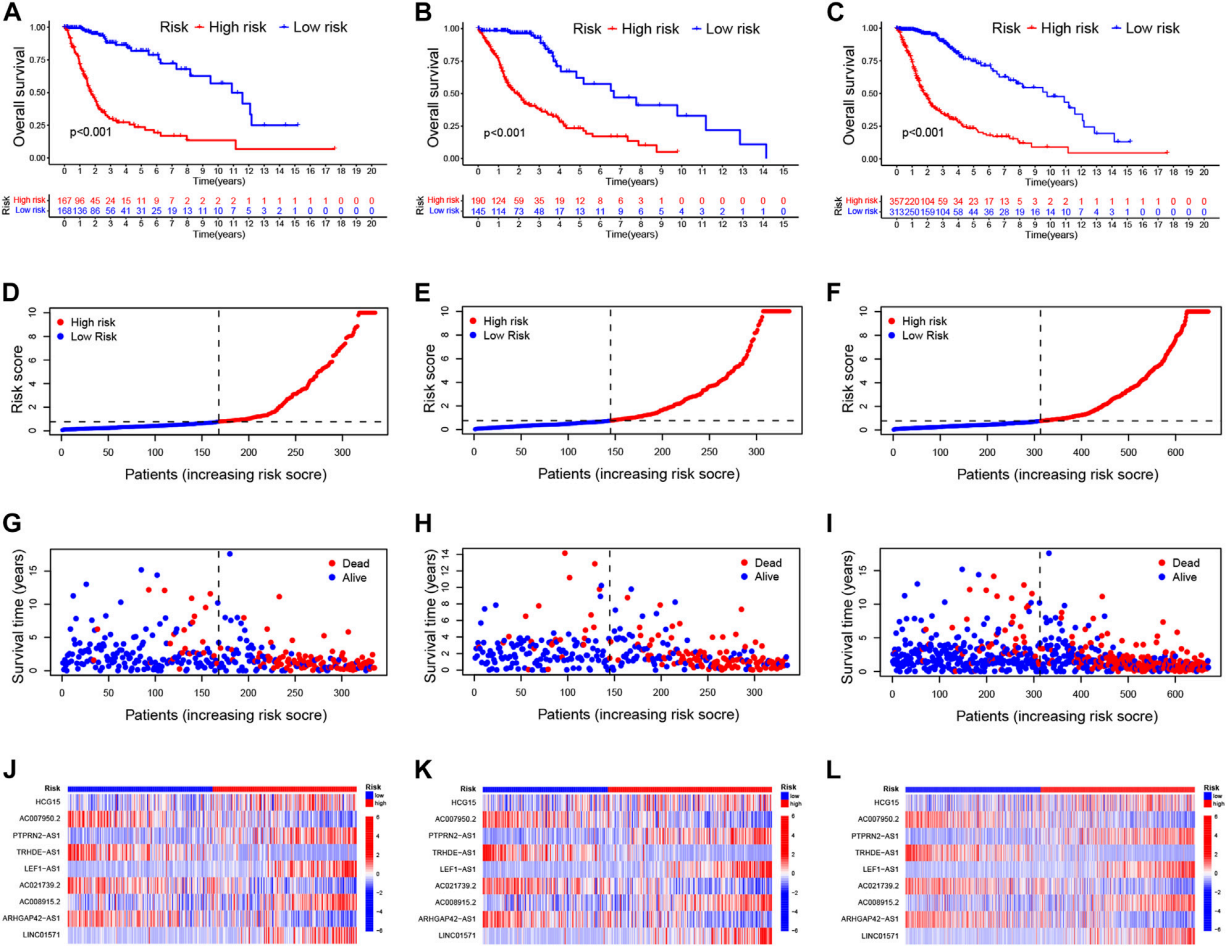
Validation of the prognostic signature of CRLs **(A–C)**. Kaplan–Meier curves for OS in the training sets **(A)**, testing sets **(B)**, and entire set **(C) (D–F)**. Distribution of the CRL-model-based risk score for the training sets **(D)**, testing sets **(E)**, and entire set **(F) (G–I)**. Patterns of survival time and survival status ranked by risk score in the training sets **(G)**, testing sets **(H)**, and entire set **(I) (J–L)**. Heatmap showing the display levels of the ten lncRNA for each patient in the training sets **(J)**, testing sets **(K)**, and entire set **(L)**. CRL, cuproptosis related lncRNA; OS, overall survival.

Patients with glioma were categorized into the high-risk and low-risk groups using the median of the risk score as a cut-off value. Using the K-M curve, OS and progression-free survival (PFS) was remarkable longer among patients in the low-risk group (Figures 2A–C; Supplementary Figure S5A). The mortality in glioma patients increases progressively with increasing risk scores (Figures 2D–I). Finally, a heatmap indicated that five CRLs were expressed higher in the high-risk group, while four others showed reversed expression (Figures 2J–L).

### 3.3 Correlation analysis between CRLs model and clinical features

**FIGURE 3:**
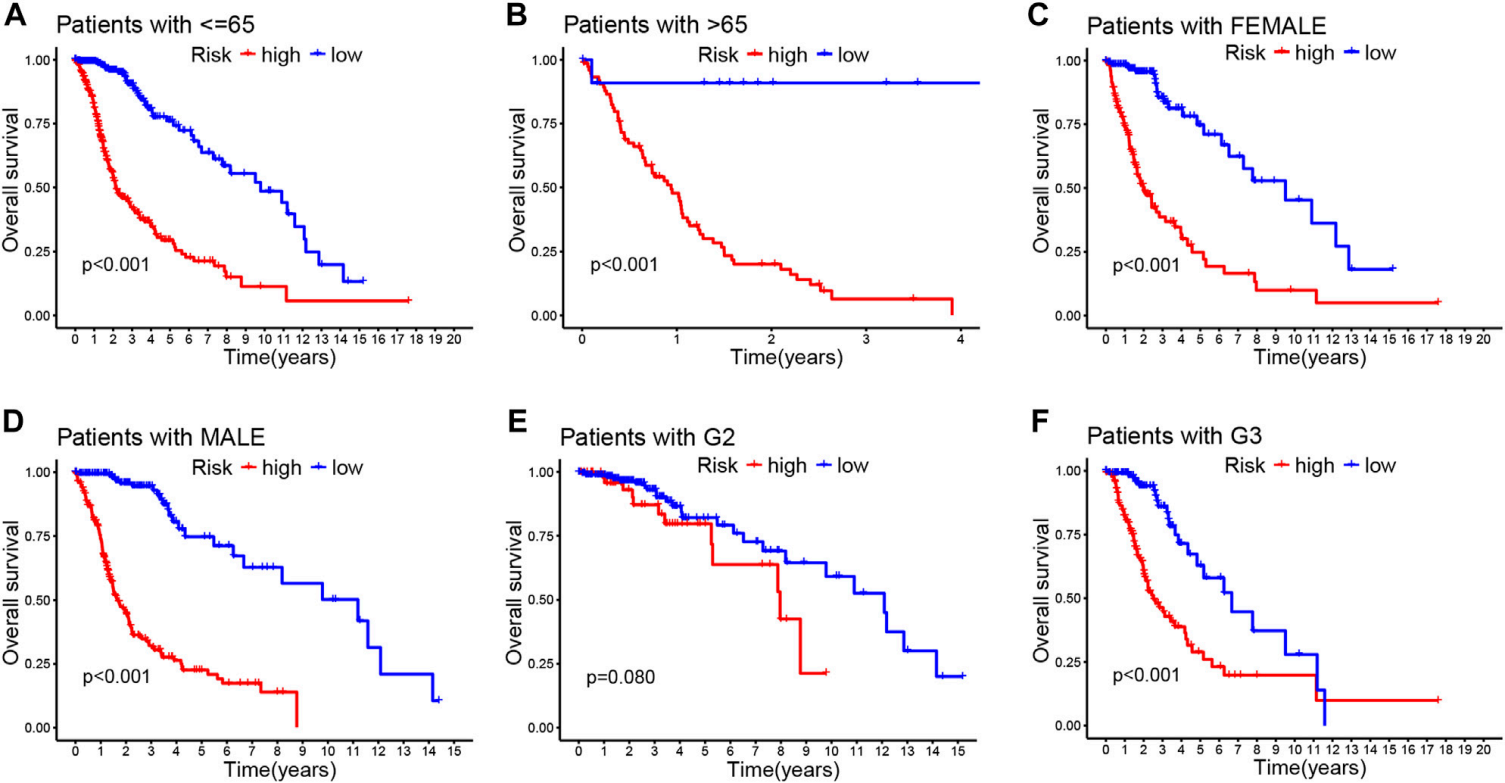
Correlation between the prognostic CRLs signature and clinicopathological characteristics **(A–F)**. Kaplan–Meier curve for overall survival in different clinical features such as age **(A,B)**, gender **(C,D)**, and tumor grade **(E,F)**.

Then, we stratified subgroups based on age, sex, and cancer grade to examine the relations between OS and risk scores. It was of great significance that the prognostic value of this risk model is dependent on the clinicopathological features of CRLs. These findings demonstrate that high-risk patients were correlated with age, sex, grade, and isocitrate dehydrogenase (IDH) as shown in Figure 3; Supplementary Figures S3A–C.

**FIGURE 4:**
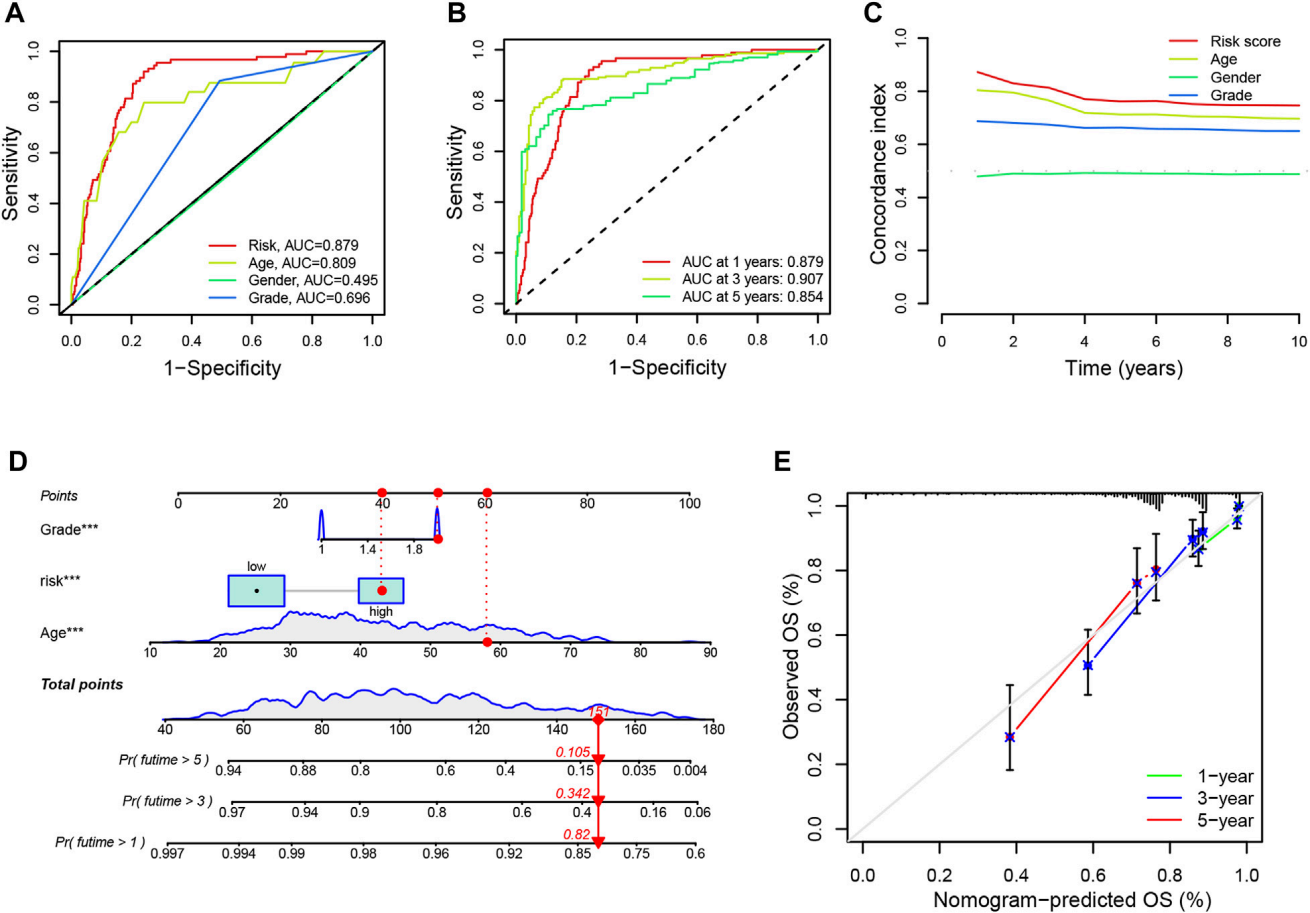
ROC curves and establishment of a nomogram **(A)**. The AUC values of the risk factors include risk scores, age, gender and tumor grade **(B)**. The AUC of the CRL signature for 1-, 3-, and 5-year survival rates in glioma **(C)**. The concordance index of the risk factors including risk scores, age, gender, and tumor grade **(D)**. A nomogram predicting the 1-, 3- and 5-year survival rates for glioma using independent prognostic factors (grade, age, and risk score) **(E)**. Calibration curves show the concordance between the prediction by nomogram and actual survival. AUC, an area under the curve; CRL, cuproptosis-related lncRNA; OS, overall survival.

Additionally, we evaluated the diagnostic value and accuracy of CRLs in glioma patients by drawing a ROC curve. The AUC of CRLs was 0.879, which was significantly higher than those other clinical features such as age (0.809), sex (0.495), and grade (0.696) (Figure 4A). In addition, AUC values for predicting 1-, 3-, and 5-year survival were 0.879, 0.907, and 0.854, respectively (Figure 4B). Overall, these findings suggest that the 9 CRLs can be relied upon to provide patients with reliable prognoses.

### 3.4 Construction and confirmation of a nomogram

Furthermore, univariate and multivariate Cox regression analyses were performed on age, gender, grade, and risk score to identify whether the signature of CRLs could be regarded as an independent prognostic indicator for OS. As seen in Supplementary Figure S3D, the model could predict OS for glioma patients (HR = 1.099; 95% confidence interval [CI], 1.076–1.122; *p* < 0.001). Following adjustment for age, gender, and grade, the risk signature was a crucial independent prognostic factor for glioma *via* multiple Cox regression (HR = 1.070, 95% CI, 1.045–1.096, *p* < 0.001) (Supplementary Figure S3E). Additionally, the prognostic model had a higher concordance index than other clinical features (such as age, sex, and grade) (Figure 4C). Besides, the nomogram was established to predict the rates of OS for patients with glioma at 1, 3, and 5 years. The results showed that the OS rates of 1, 3, and 5 years were 0.82, 0.342, and 0.105, respectively (Figure 4D). In addition, the calibration curves showed a great agreement between the predicted and actual survival (Figure 4E).

### 3.5 PCA and functional enrichment analyses

**FIGURE 5:**
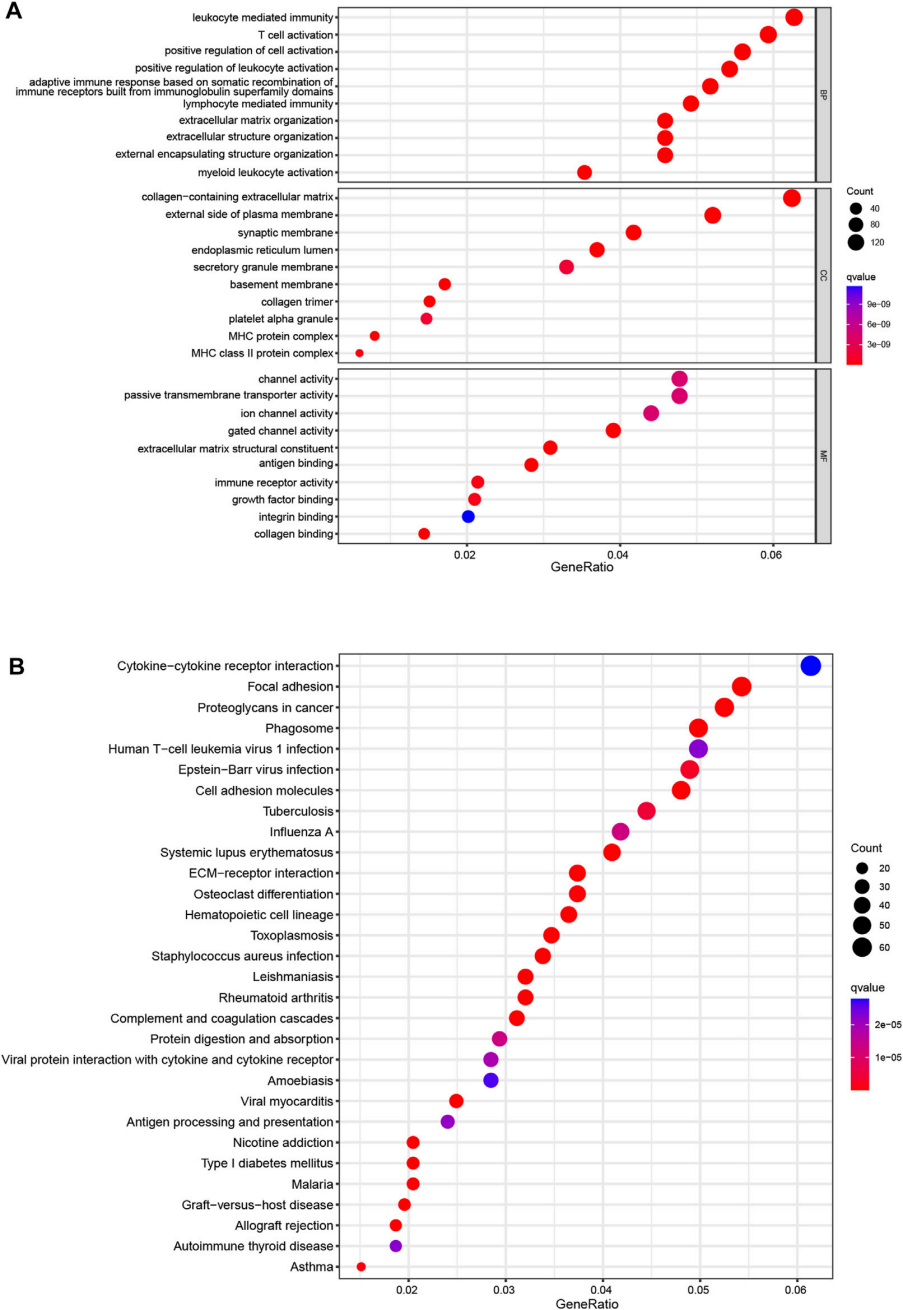
Enrichment analyses **(A)**. GO functional enrichment analysis with bubble plot (BP, biological process; CC, cellular component; MF, molecular function) **(B)**. KEGG pathway enrichment analysis with bubble plot. GO, Gene Ontology; KEGG, Kyoto Encyclopedia of Genes and Genomes.

Then, we used PCA to investigate the differences between the low- and high-risk groups. The results of PCA displayed that 9 CRL risk signature had an excellent ability to discriminate between the two groups patients (Supplementary Figures S4A–D). Next, DEGs between the two score subgroups were first explored and were then utilized to elucidate the enrichment analyses. Specifically, GO analysis demonstrated a significant enrichment for leukocyte-mediated immunity, T-cell activation, and collagen-containing extracellular matrix (Figure 5A). Simultaneously, KEGG functional analysis revealed several pathways for interactions between cells, such as cytokine–cytokine receptor interaction, focal adhesion, and cell adhesion molecules (Figure 5B). These results confirmed the involvement of DEGs in the immune responses of glioma.

### 3.6 Tumor immune microenvironment in different risk groups

**FIGURE 6:**
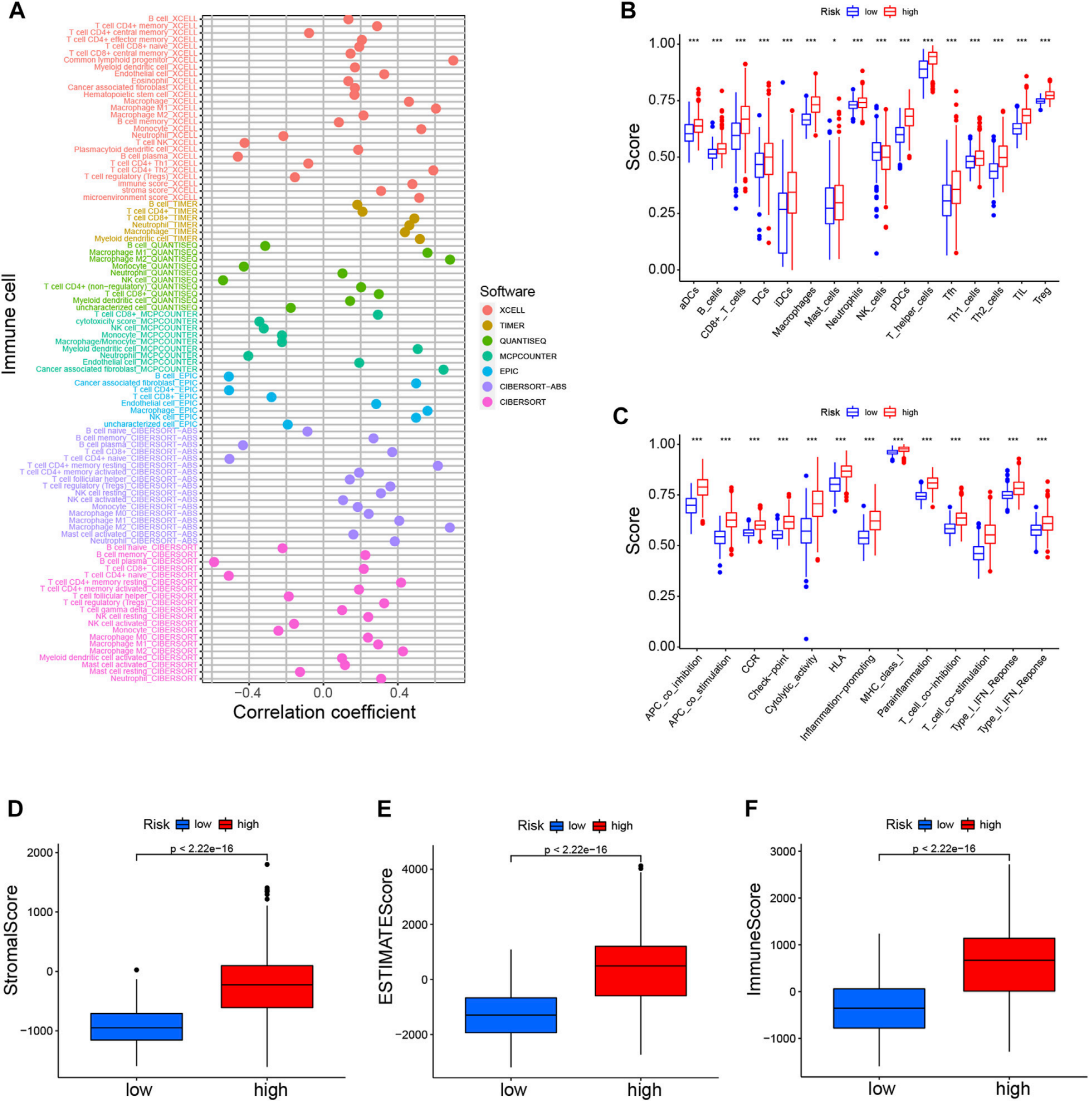
Immune landscapes of CRLs prognostic signature **(A)**. Heatmap for immune infiltration among high- and low-risk groups based on xCELL, TIMER, quanTIseq, MCP-counter, EPIC, CIBERSORT-ABS, and CIBERSORT algorithms **(B)**. SsGSEA analysis showing the extent of immune cell infiltrations in the high- and low-risk groups **(C)**. SsGSEA analysis displaying the functions of immune cell subpopulations between high-risk and low-risk groups **(D–F)**. Stromal score, ESTIMATE score, and immune score in the high- and low-risk groups. *p < 0.05, **p < 0.01, ***p < 0.001. SsGSEA, single-sample gene set enrichment analysis.

Considering the enrichment of immune-related functions in DEGs, we examined their correlation with prognostic CRLs. Several immune cells including B cells, CD4^+^ T cells, macrophages and NK cells were largely correlated with CRL scores (Figure 6A). Additionally, low-risk patients had a significantly higher NK cells counts, but a lower abundance of activated dendritic cells (aDCs), B cells, macrophages and so on (Figure 6B). Moreover, immune function scores were different in both risk groups (Figure 6C; Supplementary Figure S5B).

High-risk individuals’ immune checkpoints were expressed more strongly, which may account for the poorer OS of this group (Supplementary Figure S5C). Furthermore, we found that the various CRL-score groups exhibit different immune characteristics (Figures 6D–F). There were lower scores in the low-risk patient group for stroma, ESTIMATE, and immune function. In addition, we also explored the association between these CRLs and programmed cell death 1 ligand 1 (PD-L1). The results showed that CRLs have different degrees of correlation with PD1 or PD-L1 (Supplementary Figure S5D). In conclusion, such tumor-immune relationships in glioma could facilitate personalized immunotherapy, potentially helping to better guide the individualization of therapeutic choices.

### 3.7 TMB analysis, TIDE and therapeutic drug sensitivity evaluation of glioma

**FIGURE 7:**
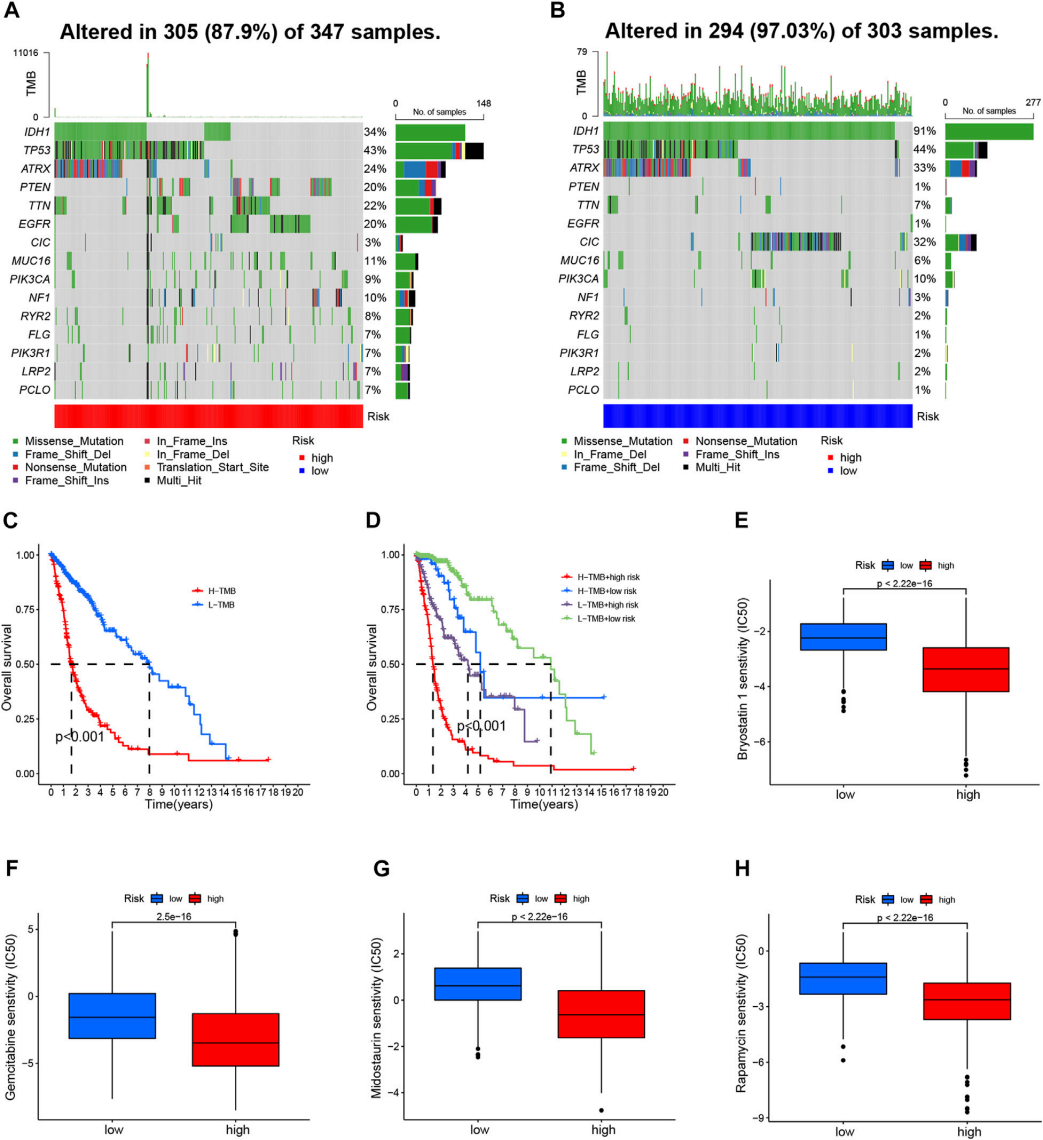
TMB analyses and drug sensitivity between high- and low-risk groups **(A, B)** Waterfall plots showing the mutational information for the genes with high mutation frequencies in the high- **(A)** and low- **(B)** risk groups **(C)**. Kaplan–Meier curve for OS of patients with glioma in high- and low-TMB groups (*p* < 0.001) **(D)**. Kaplan–Meier curve for OS of patients with glioma based on the TMB and the risk signature of CRLs **(E–H)**. The sensitivity of drugs such as bryostatin 1 **(E)**, gemcitabine **(F)**, midostaurin **(G)**, and rapamycin **(H)**, for the treatment of glioma, based on the IC50. CRL, cuproptosis-related lncRNA; OS, overall survival; TMB, tumor mutation burden.

We further compared the TMB-specific genes in the two groups. Individuals with low-risk group had mutations in the 15 genes with the highest mutation rates (Figures 7A, B). In addition, TMB scores were used to categorize glioma patients into high- and low-TMB groups. We therefore further compared the TMB in the high- and low-risk groups and observed that high-risk group had a higher TMB (Supplementary Figure S6A). Those with low TMB had a largely longer OS than those with high TMB (Figure 7C). Furthermore, TMB and CRL-scores were assessed for their synergistic effects on prognostic stratification. The most successful survivors were high-risk patients with a high TMB, while those at low risk with a low TMB had the best survival rates. Interestingly, patients of high-risk TMBs have a worse prognosis than those with low-risk TMBs regardless of the TMB status (Figure 7D). TIDE was used to investigate the sensitivity to immunotherapy for glioma patients. Nevertheless, our results revealed no significant differences between the high- and low-risk groups (Supplementary Figure S6B).

Then, we evaluated the drug sensitivity of each patient in the different groups. There was a remarkable difference in the IC50 values of bryostatin 1, gemcitabine, midostaurin, and rapamycin in the comparison between the two groups of participants (Figures 7E–H). Moreover, the IC50 values of four drugs were negatively correlated with risk scores (Supplementary Figures S6C–F).

### 3.8 Consensus cluster analysis according to prognostic CRLs

**FIGURE 8:**
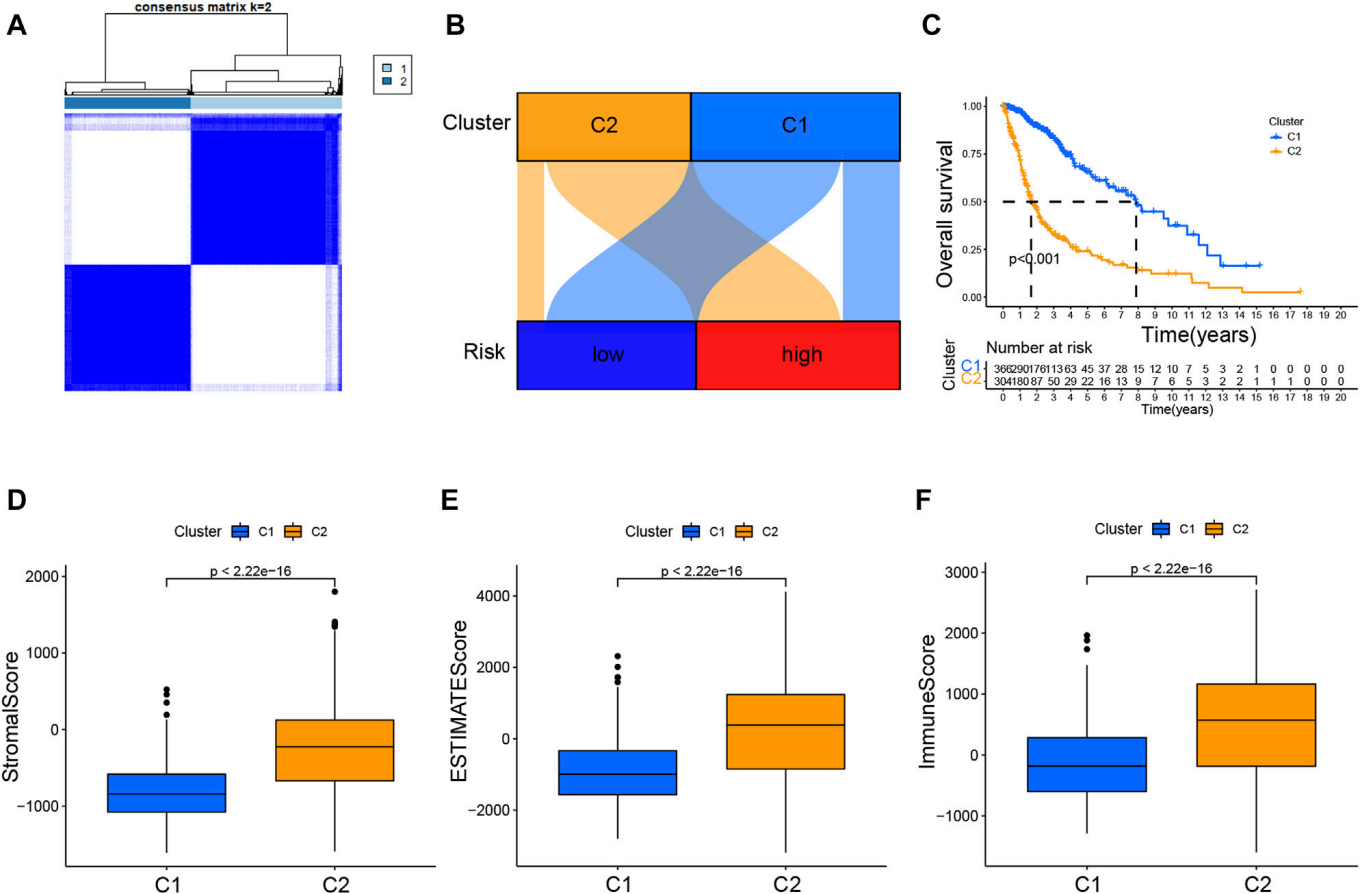
Consensus clustering analysis of CRLs and immune correlation analysis **(A)**. Consensus clustering matrix for k = 2 **(B)**. Sankey diagram showing the interaction between clusters and risk **(C)**. K–M curves for overall survival (OS) of clusters 1 and 2 **(D–F)**. Immune-related scores in clusters.

The 701 glioma samples were regrouped to compare the immune landscape of different tumor subtypes. According to the CRL signature, we grouped the patients into two clusters to conduct a consensus cluster analysis (Figure 8A; Supplementary Figure S7G). As illustrated in the Sankey diagram in Figure 8B, those with low risk were mostly categorized into cluster 1, while those with high risk were mainly grouped into cluster 2. The cluster 1 patients had a markedly longer OS than those in cluster 2 (Figure 8C). The PCA provided a clear separation of risk groups and clusters, and the tSNE confirmed statistical equality between the two groups (Supplementary Figures S7A–D).

Additionally, Supplementary Figure S7E showed the immune infiltration of two clusters was different based on various algorithms, whereas a lot of immune checkpoint genes are more expressed in cluster 2 (Supplementary Figure S7F). The results presented in the boxplots in Figures 8D–F displayed that cluster 2 had dramatically higher stromal, ESTIMATE, and immune scores.

### 3.9 Identifying LEF1-AS1 as a prognostic biomarker for glioma

**FIGURE 9:**
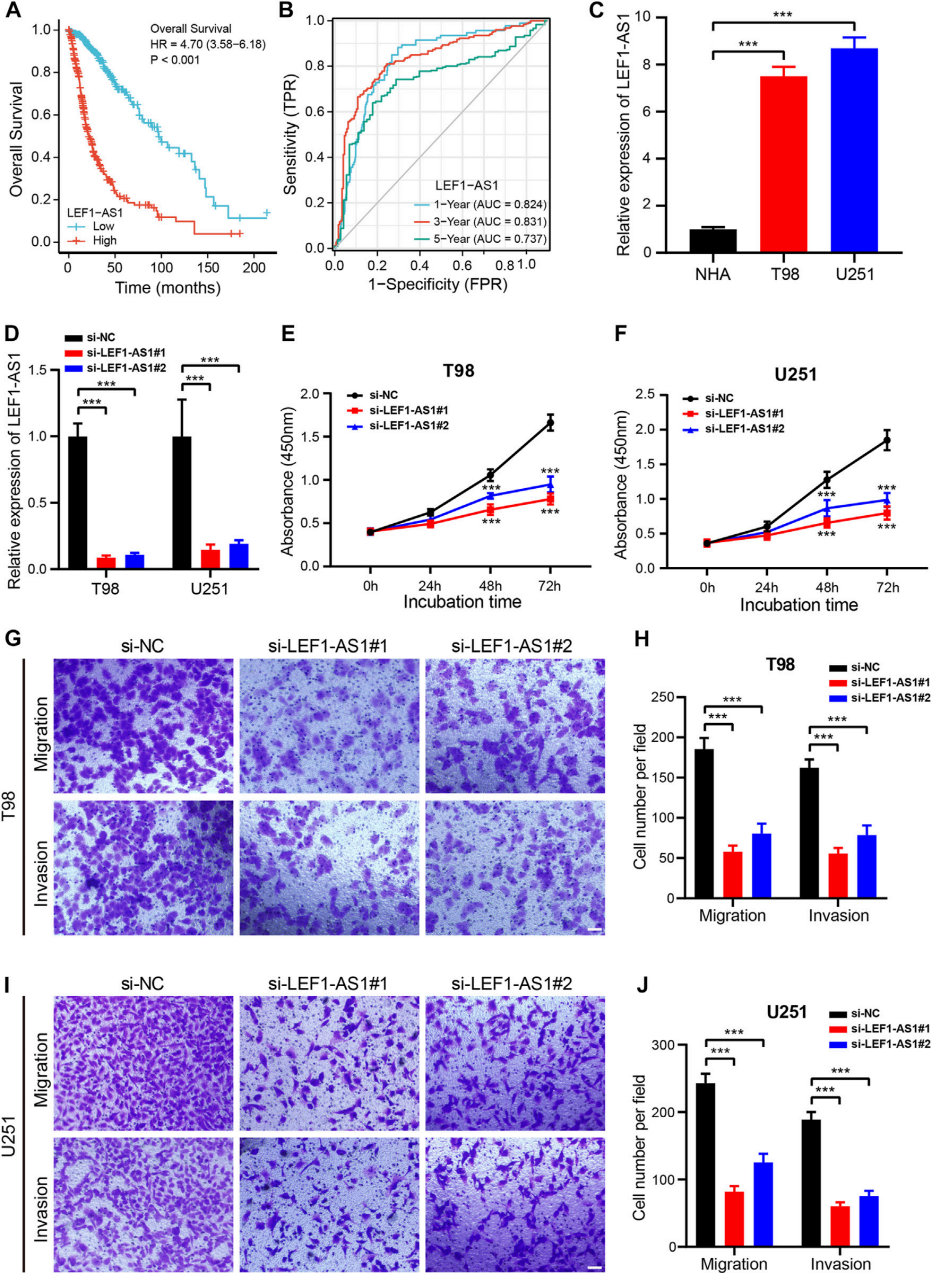
Knockdown of LEF1-AS1 inhibited cell proliferation, migration, and invasion in glioma **(A)**. Overall survival (OS) curves for glioma patients with high and low LEF1-AS1 expression **(B)**. Time-dependent ROC curves and AUC values for 1-year, 3-year, and 5-year OS prediction **(C)**. RT-qPCR analysis was applied to detect LEF1-AS1 expression in two glioma cell lines (T98 and U251) and a normal human astrocyte (NHA) cell line **(D)**. The efficiency of si-LEF1-AS1 transfection in T98 and U251 cells was assessed by RT-qPCR **(E–F)**. Cell proliferation of T98 **(E)** and U251 **(F)** cells transfected with control (si-NC) or si-LEF1-AS1 was measured *via* CCK8 assay **(G–J)**. Migratory and invasive capacities of LEF1-AS1-deficient T98 **(G,H)** and U251 **(I,J)** cells were determined using Transwell cell migration and invasion assays. Scales bar, 100 μM. ***p < 0.001.

As a further step toward identifying a prognostic biomarker for glioma related to cuproptosis, we examined the expression level of the 9 CRLs created to formulate the prognostic signature for glioma using the TCGA dataset. HCG15, AC007950.2, PTPRN2-AS1, LEF1-AS1, AC021739.2, ARHGAP42-AS1, and LINC01571 were found to be highly expressed in glioma tissues, while TRHDE-AS1 and AC008915.2 were highly expressed in normal cells (Supplementary Figure S8). Low LEF1-AS1 expression patients had remarkably longer OS and PFS (Figure 9A; Supplementary Figure S8B). Additionally, the AUCs values of LEF1-AS1 at 1 year, 3 years, and 5 years were 0.8244, 0.8311, and 0.737, respectively (Figure 9B). Moreover, we further assessed the potential of LEF1-AS1 as a prognostic predictor for glioma patients using the CGGA (mRNAseq_325 and mRNAseq_693) dataset and the results were consistent with those from the TCGA database (Supplementary Figure S9), suggesting that LEF1-AS1 level was highly predictive of the prognosis of patients.

### 3.10 Inhibition of LEF1-AS1 prevented cell proliferation, migration, and invasion in glioma

Our first step was to test the LEF1-AS1 expression level by RT-qPCR in two glioma cell lines (T98 and U251). The LEF1-AS1 was considerably less expressed in NHA cells than in T98 and U251 cells (Figure 9C). Then, we inhibited LEF1-AS1 using two siRNAs in the glioma cell lines and RT-qPCR displayed that two independent siRNAs effectively silenced LEF1-AS1 (Figure 9D). The growth curves displayed that depletion of LEF1-AS1 remarkably impaired the growth of glioma cells (Figures 9E, F). Besides, a transwell assay was conducted to confirm whether LEF1-AS1 inhibition affects glioma cells’ migration and invasion abilities. We confirmed that LEF1-AS1 inhibition prevented T98 (Figures 9G, H) and U251 (Figures 9I, J) cells from migrating and invading. Collectively, these findings hinted that LEF1-AS1 is related to the promotion of glioma cell growth and migration *in vitro* and it could be a future treatment target for glioma.

## 4 Discussion

Glioma patients frequently experience dismal outcomes and elevated mortality rates (Yang et al., 2022a). The persistent problems of unexpected recurrence and inadequate survival plague individuals suffering from glioma (Yang et al., 2022b). Despite the advancements made in survival rates, the prognosis for glioma patients remains unfavorable. Recently, researchers have devised a CRLs-based risk model to forecast treatment outcomes for glioma patients, which indicates the potential significance of CRLs in future glioma therapy (Wang et al., 2022). To illustrate, certain scholars have constructed and confirmed a new risk model using CRLs to determine the therapy response and prognosis of glioma patients (Zhang et al., 2022c; Wu et al., 2023). However, no specific CRLs biomarkers have been identified for glioma therapy and prognosis.

We probed both the role and value of CRLs in glioma using comprehensive analyses. First, based on 9 CRLs associated with glioma prognosis, we created and verified the prognostic signature for glioma. Regardless of the training or testing set, OS was remarkable longer in the low-risk patients. Next, we confirmed two molecular subtypes of glioma using consensus cluster analysis, with the cluster one subtype exhibiting a remarkably longer OS than the cluster two subtype. Additionally, our team examined the involvement of immune infiltration in glioma and potential drugs for glioma therapy based on the prognostic signature. Finally, we identified the CRL LEF1-AS1 as being important and validated its role in glioma, as knockdown of the protein inhibited cell growth, migration, and invasion.

According to the emerging evidence, immune cell infiltration has been a critical element in glioma progression and significantly influences patients’ survival (Yu et al., 2021). In the current study, DEGs exhibited an enhanced affinity for immune response-related biological processes such as leukocyte-mediated immunity, and cytokine–cytokine receptor interaction. These results suggest that tumor-infiltrating immune cells are primarily responsible for creating the tumor microenvironment, which is significantly related to glioma progression. In addition, Wang et al. found that using NK cells might be a hopeful approach to treating the glioma patients (Wang et al., 2021). Our study revealed NK cells were found in large numbers in the low-risk group, linking the CRL signature to both a good prognosis and efficacious glioma treatment. An immunomodulatory molecule called PD-1 is an extremely negative factor, and its ligands include PD-L1 and PD-L2 (Ribas and Wolchok, 2018). By attaching to PD-1 on the surface of activated T lymphocytes, PD-L1 on the surface of tumor cells reduces T cell-mediated cytotoxicity and promotes the proliferation and immune escape of the tumor cells (Litak et al., 2019). Furthermore, immune checkpoint-associated gene expression differed markedly between the two risk groups, meaning our work might provide a promising regimen for identifying glioma patients who may gain more from the blockade of immune checkpoint. These results might add a new and accurate immunotherapeutic approach for the glioma patients.

To our knowledge, a major obstacle to improving the prognosis of the patients of glioma may be the incomplete and poor understanding of glioma heterogeneity and its TMB characteristics in therapeutic intervention (Wu et al., 2018); therefore, we subsequently conducted a TMB analysis. TMBs of 15 genes with the highest mutation rates were largely different for the two risk groups. Besides, several research reported that IDH1 mutation gliomas were likely to respond well to radiation and alkylating chemotherapy (Yan et al., 2009; Wang et al., 2015). Additionally, patients of low-risk group had better prognoses regardless of TMB status, indicating the CRL signature was highly predictive of the glioma patients’ prognosis even with other confounding factors. As evidence suggests, the TIDE algorithm was performed to predict the clinical effectiveness of patients to immune checkpoint inhibitors (ICI) therapy. A higher TIDE score indicates greater likelihood of immune escape, which implies that durable responses might still not be seen in ICI-treated patients. However, no significant association was observed in TIDE between the high- and low-risk groups in our study. This phenomenon may be caused by the biased data from the TCGA database or the tissue heterogeneity. The mechanisms remain unproven and more research are warranted to definite its mechanisms.

Drug sensitivity testing is crucial for determining how effective some chemotherapy agents are. It has been noticed that bryostatin 1 can increase the radiosensitivity of malignant gliomas and make them more sensitive to certain chemotherapeutic drugs (Dagur et al., 2015). Gemcitabine, a nucleoside analog, was reported to inhibit the elongation of the DNA chain and can be used as an agent for the therapy of various tumors, especially glioma (Morfouace et al., 2014). Bastiancich et al. found that gemcitabine can be a powerful radiosensitizer that could increase antitumor immune activity (Bastiancich et al., 2018). Rapamycin has been discovered to have substantial anti-neoplastic activity in GBM and affects the glioma growth and proliferation (Cloughesy et al., 2008). Another drug, midostaurin, has shown promising efficacy in the immunomodulatory process in some tumors, such as colon cancer, ovarian cancer, and glioma (Stone et al., 2017; Ke et al., 2022; Lai et al., 2022). Our study indicated that the IC50 values of these four drugs were markedly higher in patients with low-risk glioma, hinting CRL signature could add promising value for predicting the treatment response in glioma.

In line with our results, other research groups have built a CRLs signature for predicting glioma prognosis and immunotherapy response (Yan et al., 2022a; Wang et al., 2022; Xu et al., 2022). However, what we have learned about the contribution of lncRNAs to cuproptosis is only the tip of the iceberg. As far as we know, specific biomarkers for the treatment of gliomas based on CRLs have never been reported. In our current study, the CRL prognostic signature included 9 CRLs, which have been related to glioma growth and progression (Yuan et al., 2022). HCG15 (human leukocyte antigen complex group 15) facilitates proliferation and invasion by enhancing ZNF641 transcription in hepatocellular carcinoma (Yan et al., 2022b). A study has shown that TRHDE-AS1 is a low-expression gene in lung cancer and overexpression of TRHDE-AS1 affects the cell growth through the miRNA-103/KLF4 axis (Zhuan et al., 2019). A study has shown that LEF1-AS1 regulates the growth and migration of hypopharyngeal squamous cell carcinoma cells (Fan et al., 2022). Additionally, Cheng et al. (2020) demonstrated that LEF1-AS1 promotes glioma tumorigenesis by sponging miR-489-3p. Currently, few studies have investigated whether these 9 lncRNAs contribute to the progression of gliomas.

To confirm whether the 9 CRLs could be prognostic markers for glioma, we examined their expression levels using TCGA dataset. HCG15, AC007950.2, PTPRN2-AS1, LEF1-AS1, AC021739.2, ARHGAP42-AS1, and LINC01571 were highly expressed in glioma tissues. The low LEF1-AS1 expression patients had remarkably longer OS and PFS, suggesting that LEF1-AS1 level was highly predictive of the prognosis of glioma. Next, LEF1-AS1 was considerably less expressed in NHA cells than in T98 and U251 cells. Depletion of LEF1-AS1 remarkably impaired the growth of glioma cells. Moreover, we confirmed that LEF1-AS1 inhibition prevented T98 and U251 cells from migrating and invading. These findings hinted that LEF1-AS1 is associated with promotion of cell growth and migration *in vitro* and it could represent a useful therapeutic target of glioma patients.

Nevertheless, this study had some limitations. First, because of the intrinsic restriction of bioinformatics analysis in predicting drug sensitivity, prospective validation studies are needed to confirm our predictions for medication sensitivity. Then, the exact molecular mechanism by which LEF1-AS1 affects glioma growth, migration, and invasion has not been entirely clarified. Our future studies will examine the regulatory mechanism of LEF1-AS1 *in vitro* and *in vivo*.

## 5 Conclusion

CRL signatures were verified in this study as a prognostic and treatment response indicator for glioma patients. LEF1-AS1 inhibition can prevent the growth, migration, and invasion of gliomas. LEF1-AS1 may be an effective prognostic biomarker and treatment for glioma.

## Data Availability

The datasets presented in this study can be found in online repositories. The names of the repository/repositories and accession number(s) can be found in the article/Supplementary Material.

## Abbreviations

TCGA, The Cancer Genome Atlas; LASSO, Least absolute selection operator; CLRs, Cuproptosis-related lncRNAs; OS, Overall survival; PFS, Progression-free survival; ROC, Receiver operating characteristic; AUC, Area under the receiver operating characteristic; CDF, Cumulative distribution function; PCA, Principal component analysis; DEGs, Differentially expressed genes; GO, Gene Ontology; KEGG, Kyoto Encyclopedia of Genes and Genomes; TMB, Tumor mutation burden; TIDE, tumor immunity dysfunction and exclusion algorithm; CDF, cumulative distribution function.
