# Supplementary figures and images for: A novel signature of cuproptosis-related lncRNAs predicts prognosis in glioma: Evidence from bioinformatic analysis and experiments
Source: Front Pharmacol. 2023 Apr 10;14:1158723. doi: 10.3389/fphar.2023.1158723 (PMC10123286; doi:10.3389/fphar.2023.1158723)

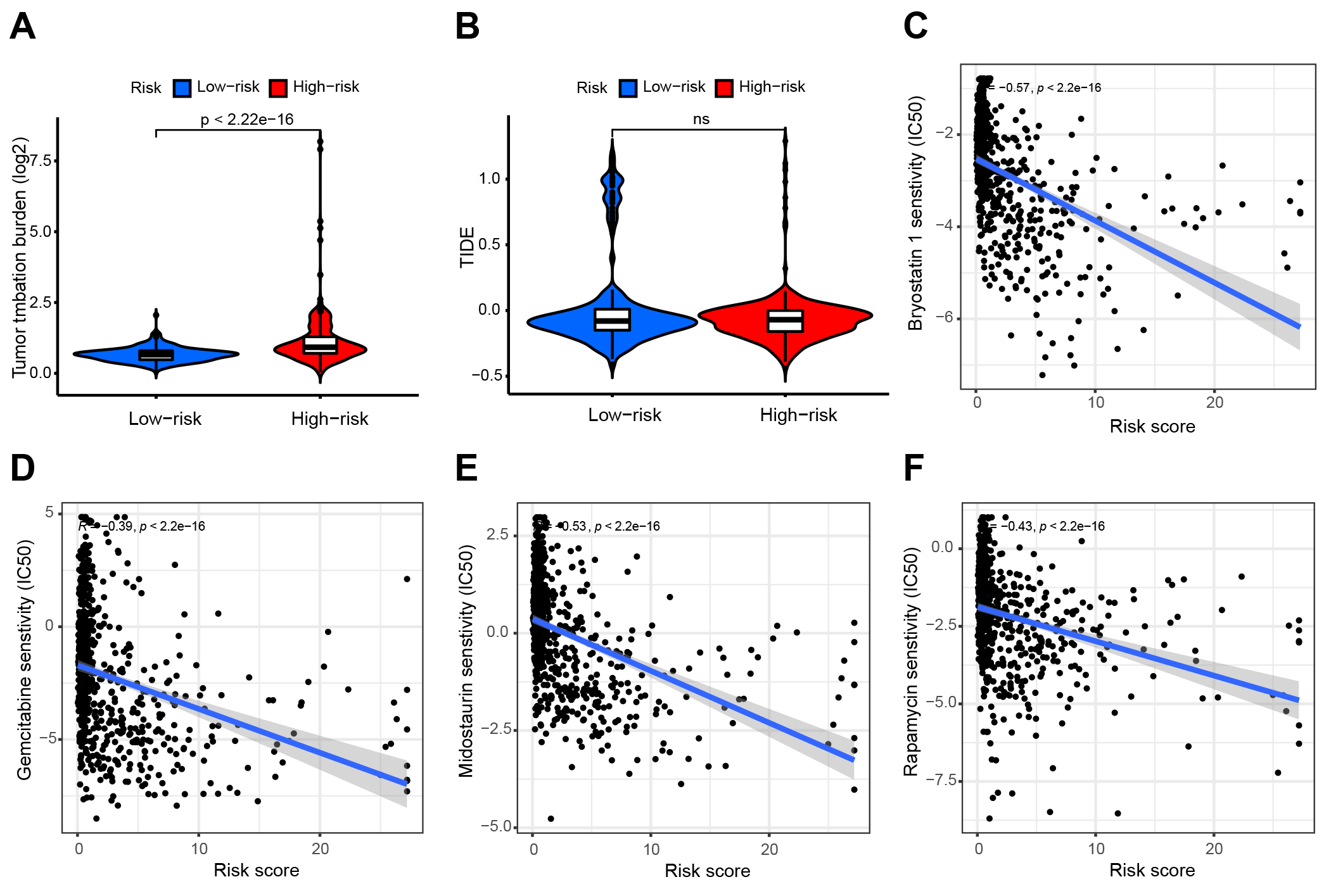

Supplement: Supplementary file 1 [file Image6.TIF]

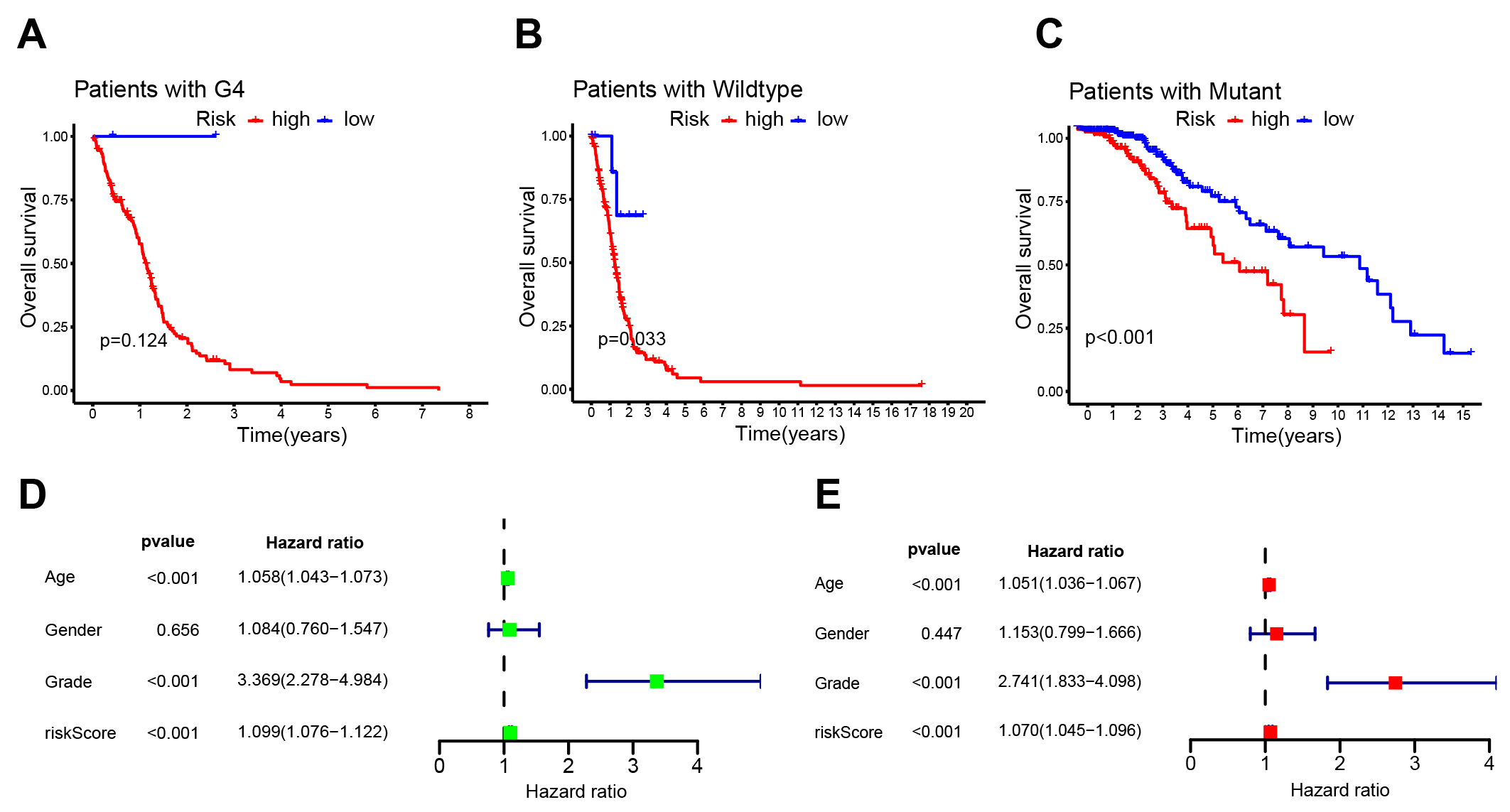

Supplement: Supplementary file 2 [file Image3.TIF]

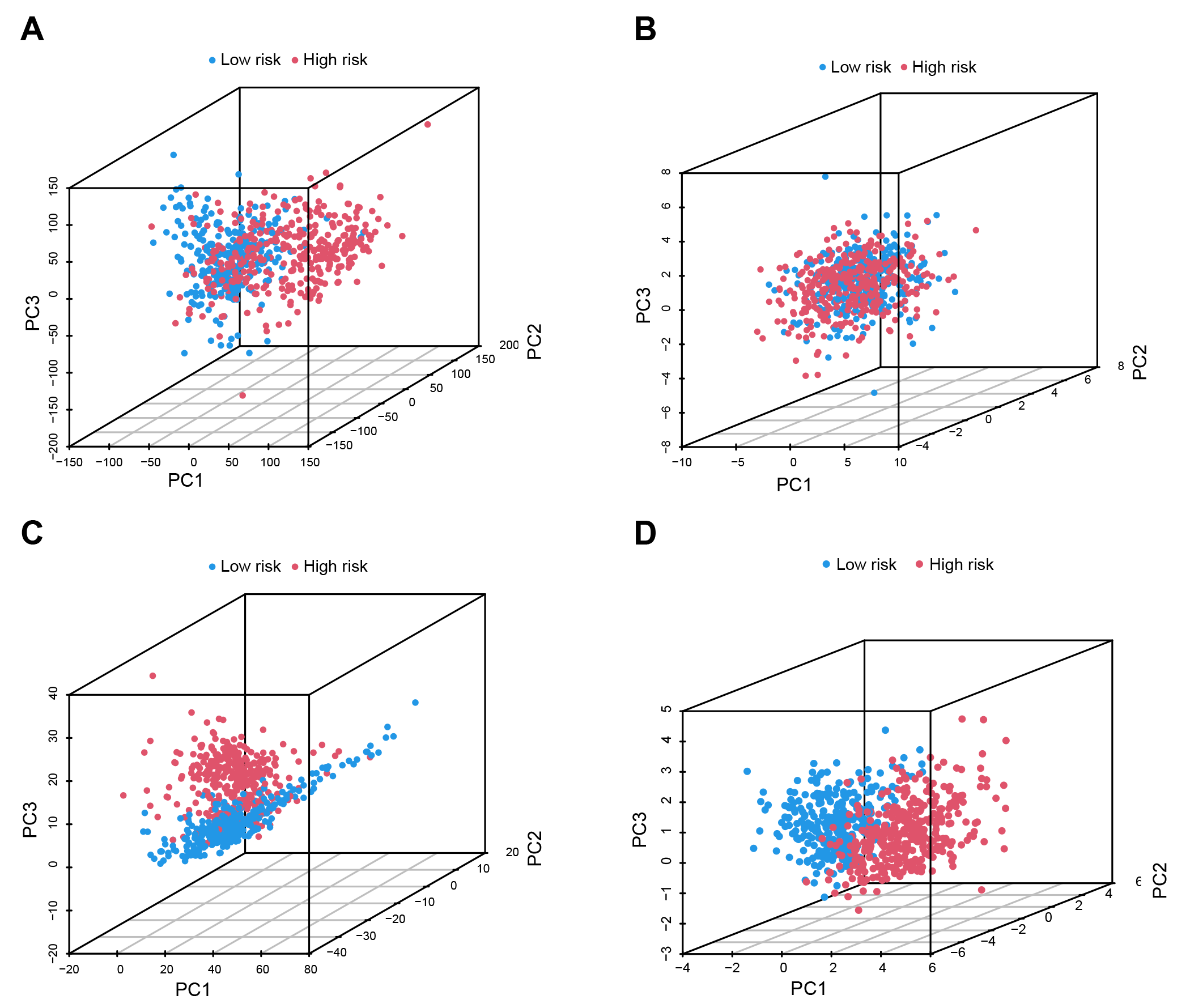

Supplement: Supplementary file 3 [file Image4.TIF]

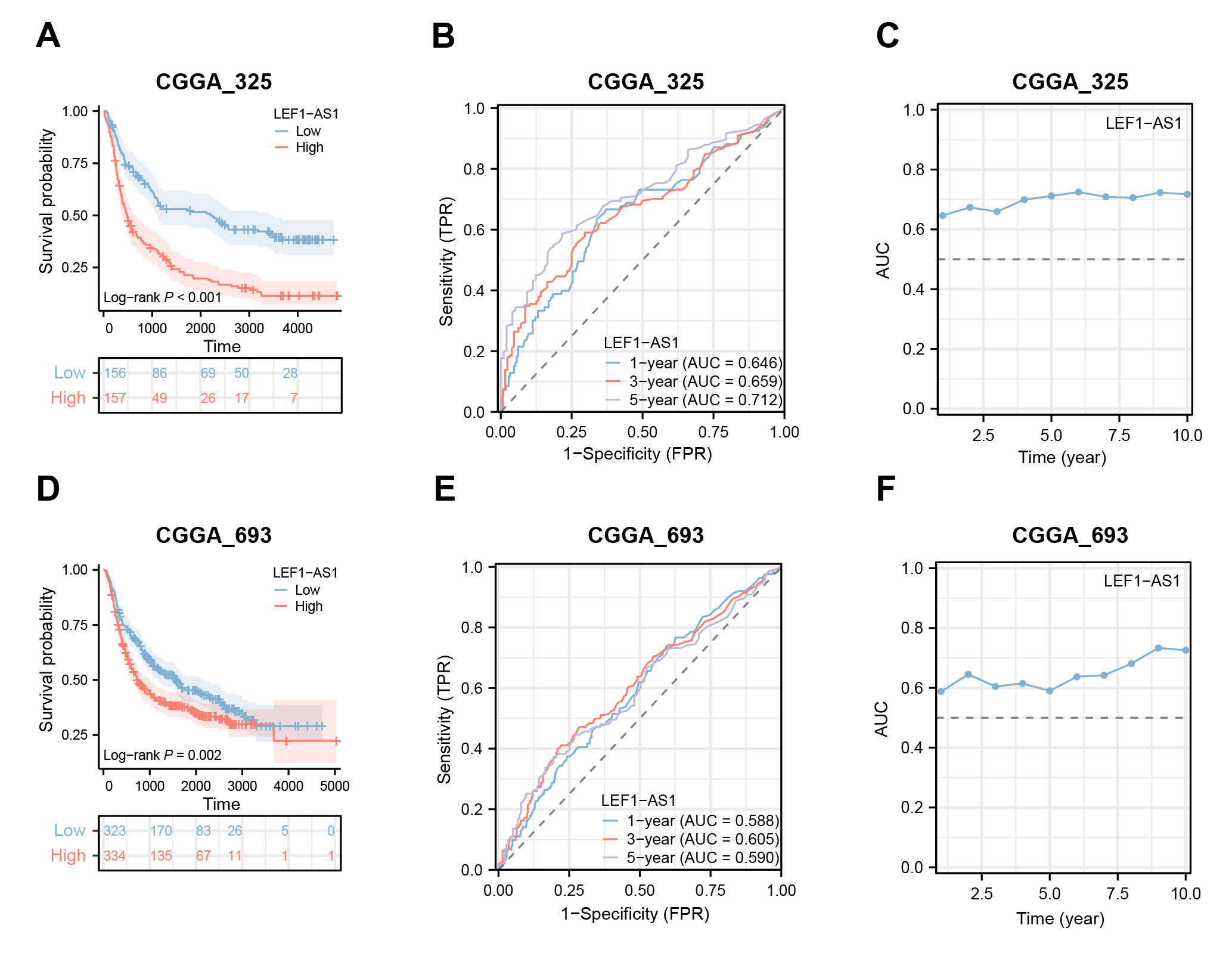

Supplement: Supplementary file 4 [file Image9.TIF]

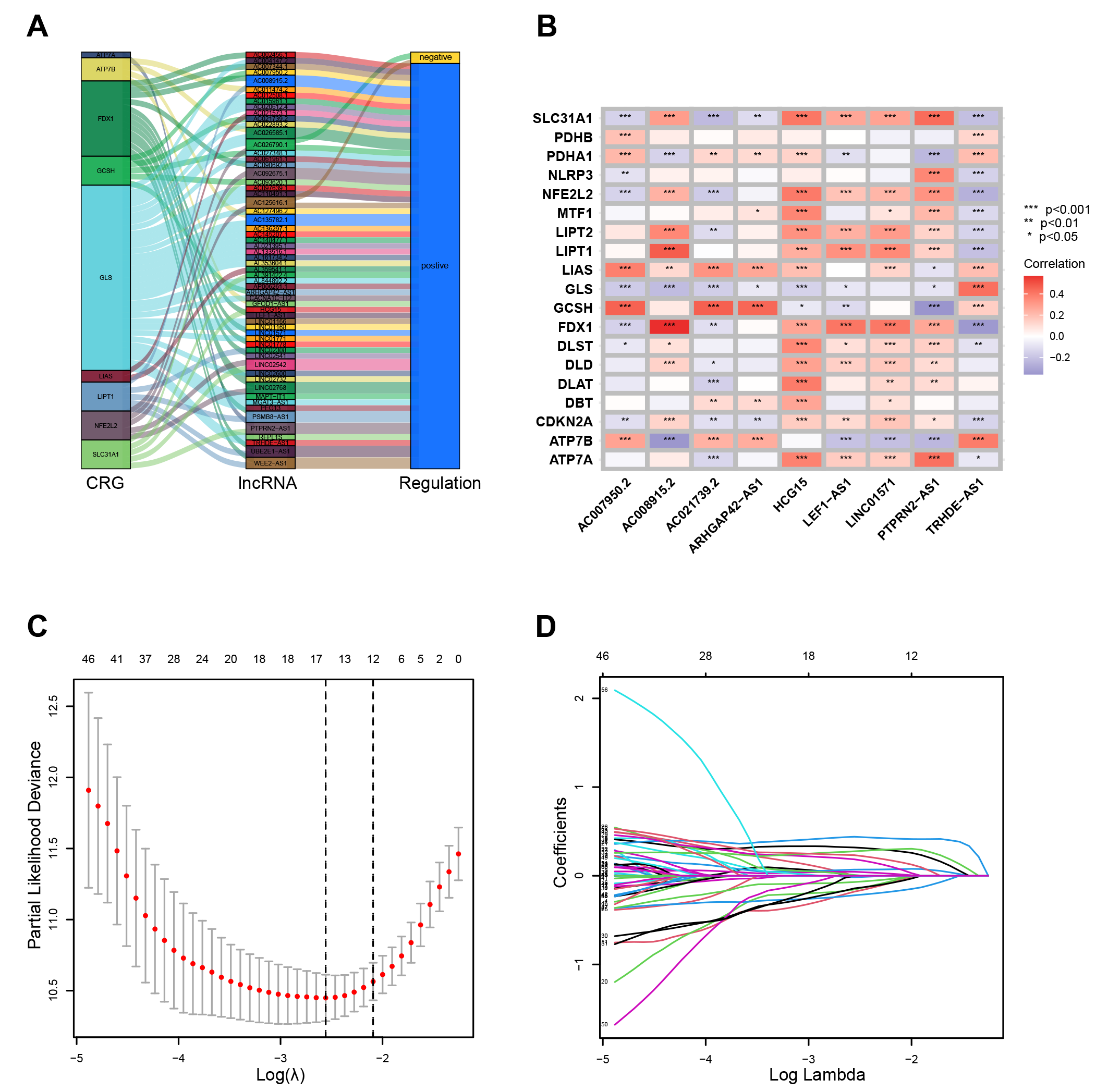

Supplement: Supplementary file 5 [file Image2.TIF]

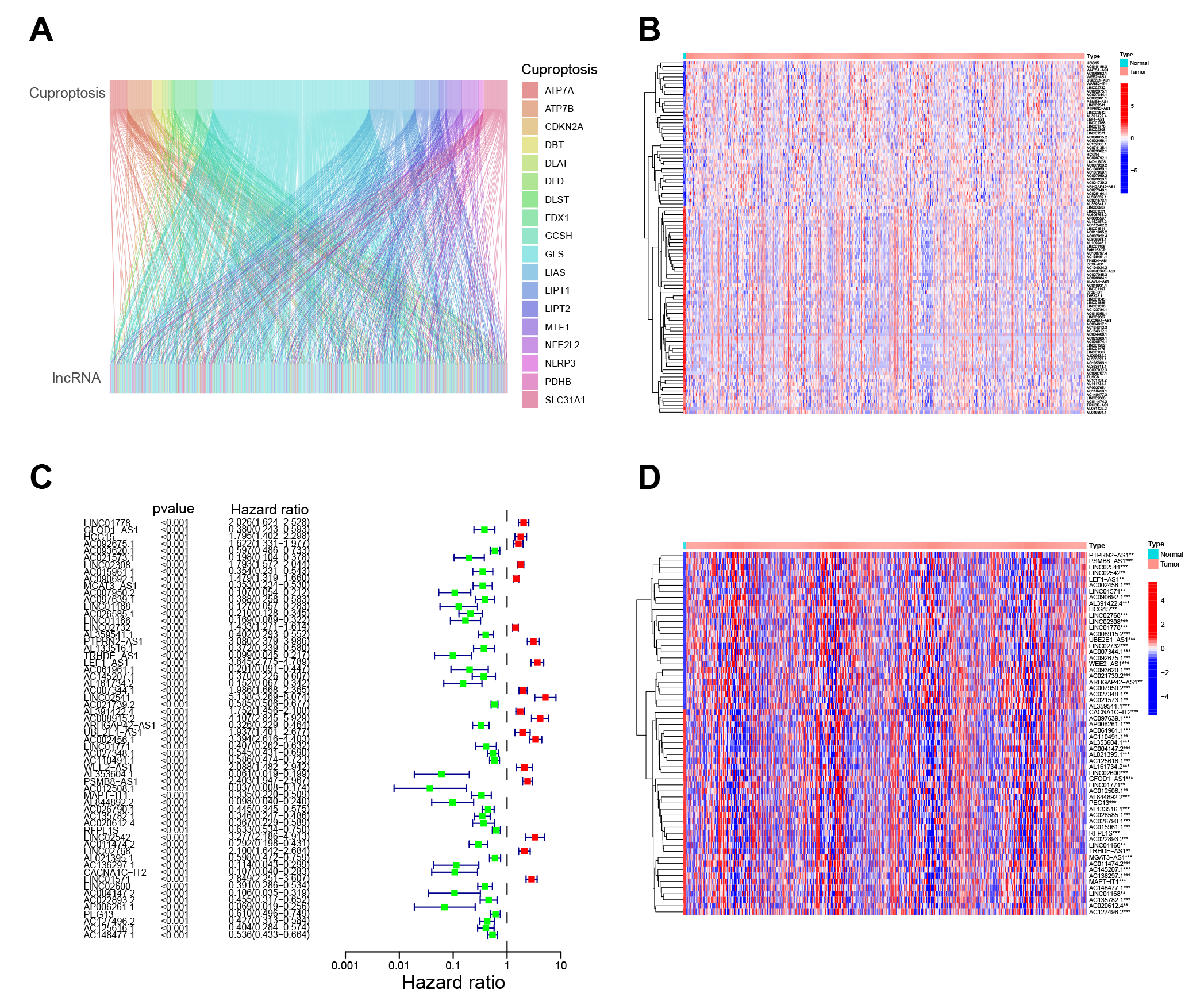

Supplement: Supplementary file 6 [file Image1.TIF]

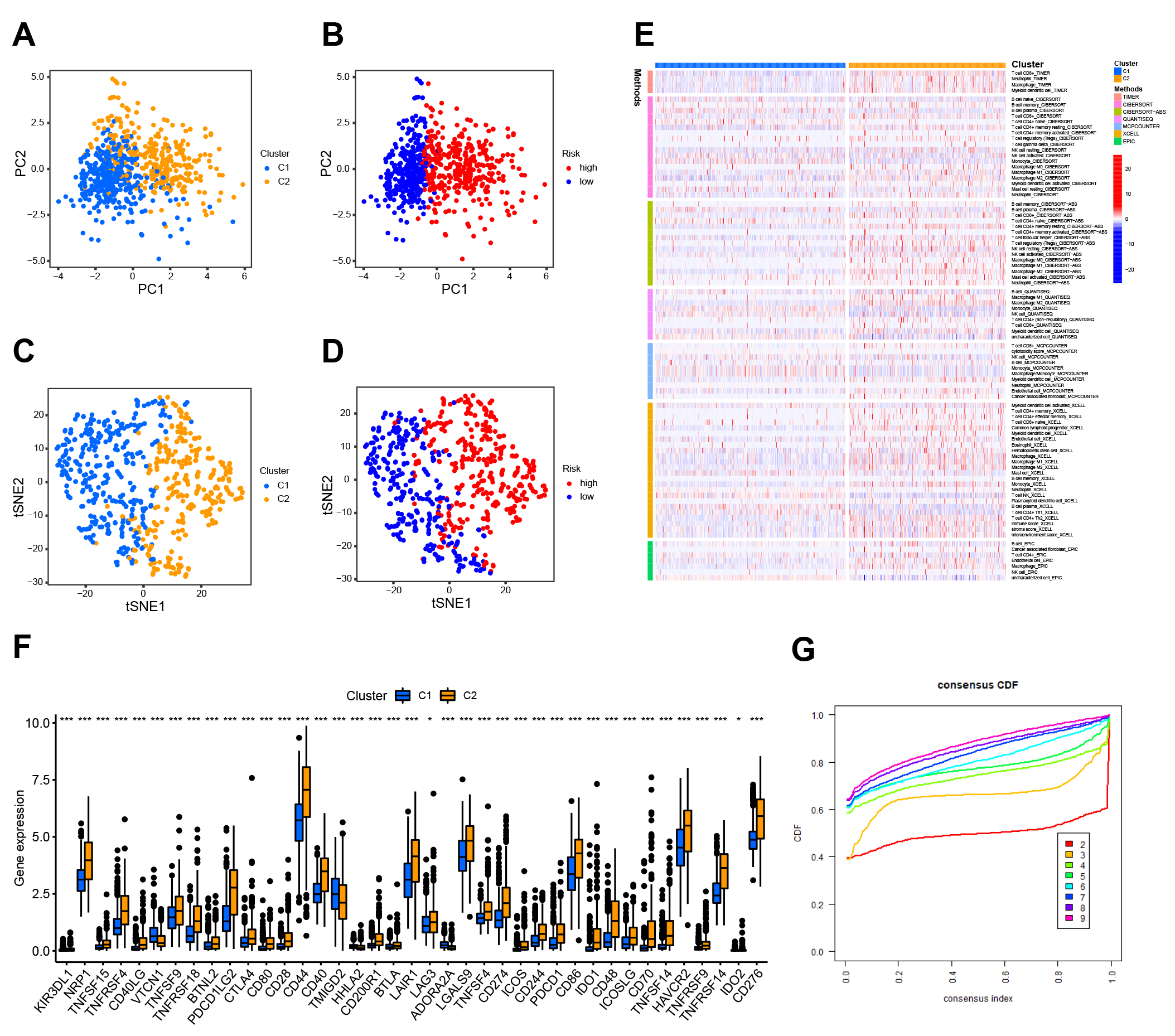

Supplement: Supplementary file 7 [file Image7.TIF]

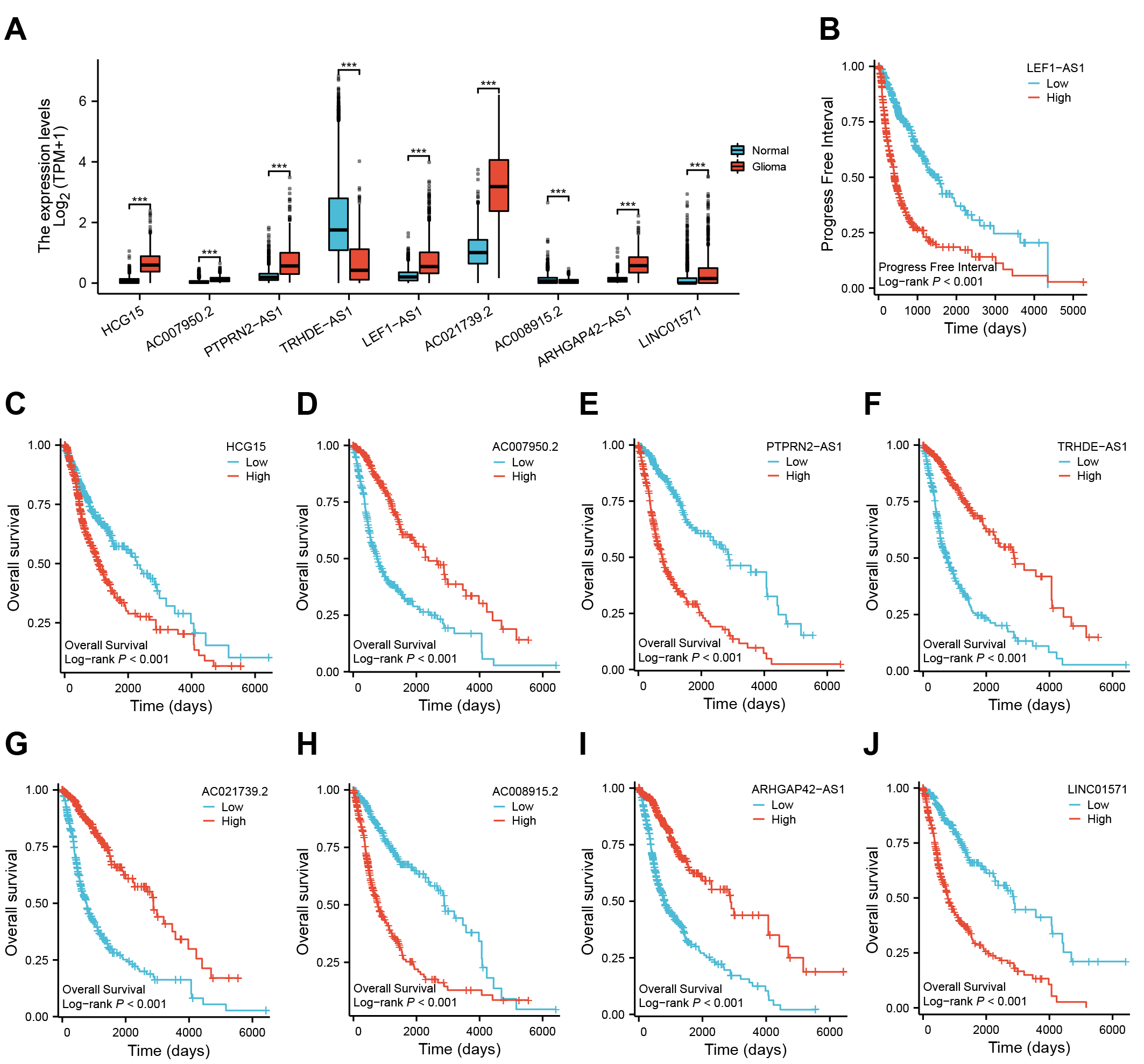

Supplement: Supplementary file 8 [file Image8.TIF]

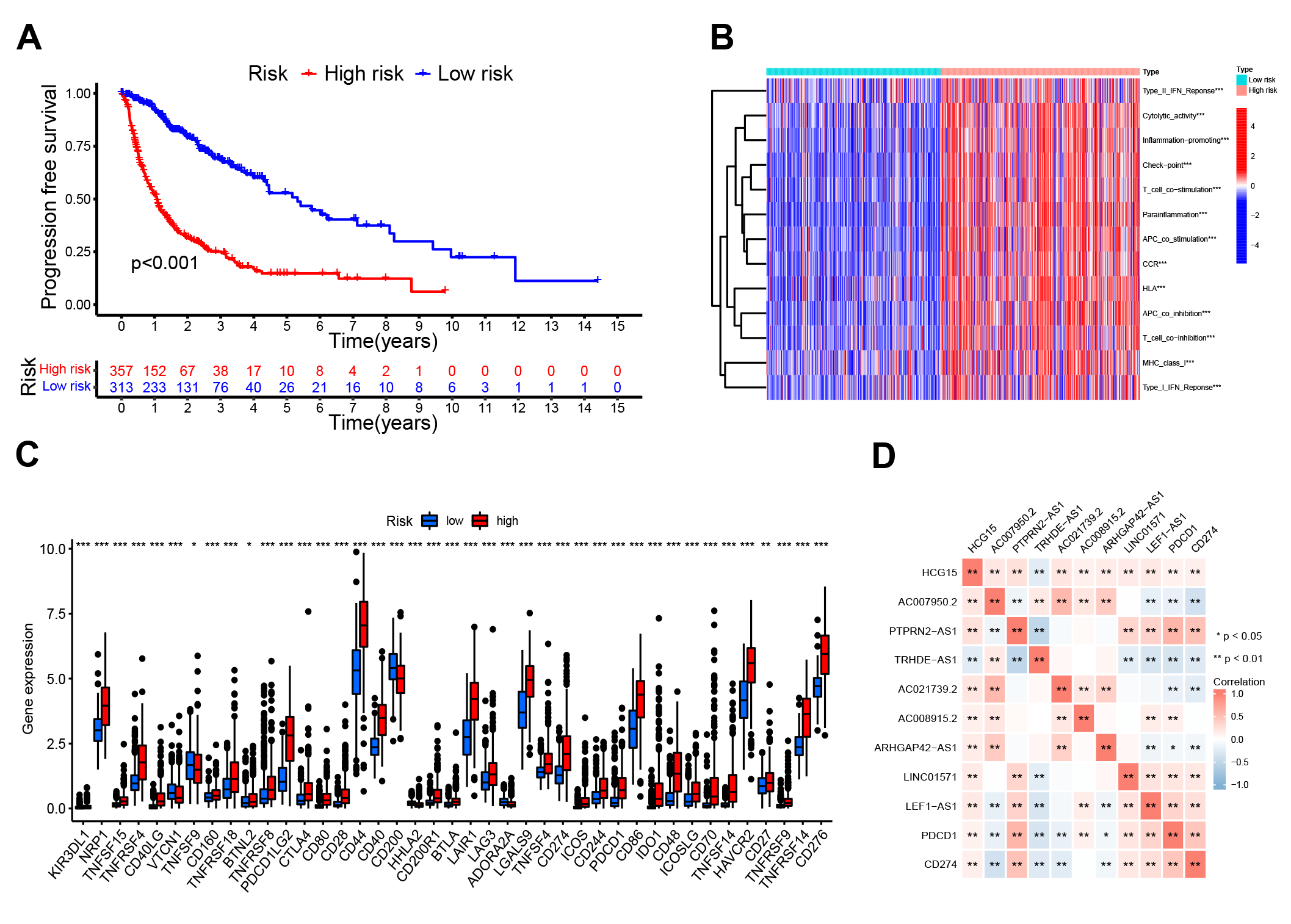

Supplement: Supplementary file 9 [file Image5.TIF]
