# Supplementary material for: A novel signature of cuproptosis-related lncRNAs predicts prognosis in glioma: Evidence from bioinformatic analysis and experiments
Source: Front Pharmacol. 2023 Apr 10;14:1158723. doi: 10.3389/fphar.2023.1158723 (PMC10123286; doi:10.3389/fphar.2023.1158723)
Supplement: Supplementary file 10 [file DataSheet1.docx]

**Supplementary figure legends**

**Figure S1. Identification of prognostic CRLs in patients with glioma by univariate analysis. (A).** Sankey diagram showing the interaction between cuproptosis-related genes and CRLs (Pearson correlation analysis, |Pearson R| > 0.4 and p < 0.001). **(B).** Heatmap showing the differential expression of CRLs between normal and tumor samples (|log2 fold change (FC)| > 2 and p < 0.005). **(C).** Forest plot of univariate analysis results showing 60 OS-related CRLs (p < 0.001). **(D).** Heatmap showing the expression of 60 OS-related CRLs in the normal and tumor samples. CRL, cuproptosis related lncRNA.

**Figure S2. Identification of the cuproptosis-related gene by Lasso cox regression analysis in glioma. (A).** Sankey diagram showing the interaction between cuproptosis-related genes and 60 OS-CRLs. **(B).** Heatmap showing the relationship between cuproptosis-related genes and 9 CRLs. **(C).** Cross-validation plot for the penalty term. **(D).** Diagram for LASSO expression coefficients.

**Figure S3. An independent prognostic analysis of clinical parameters and risk scores. (A-C)** Kaplan–Meier curve for overall survival in different clinical features such as tumor grade **(A) and** IDH mutation **(B, C)**. **(D).** Univariate Cox regression analysis of the clinical characteristics and risk score with the OS. **(E).** Multivariate analysis of the clinical characteristics and risk score with the OS. *p < 0.05, **p < 0.01, ***p < 0.001. CRL, cuproptosis related lncRNA; LASSO, least absolute selection operator; OS, overall survival.

**Figure S4. PCA functional enrichment analyses.** **(A–D).** PCA showing the distribution differences between the high- and low-risk groups according to the entire gene expression **(A)**, cuproptosis genes **(B)**, CRLs **(C),** and the risk signature of four CRLs **(D)**. PCA, principal component analysis; CRL, cuproptosis related lncRNA.

**Figure S5. The immune infiltration of functions of immune cell subpopulations and the expression of immune checkpoint genes in high and low risk patients with glioma.** **(A).** Kaplan–Meier curves for progression-free survival (PFS) in the high- and low-risk groups. **(B).** Heatmap displaying the functions of immune cell subpopulations between high-risk and low-risk groups. **(C).** The expression of immune checkpoint genes between high- and low-risk groups. **(D).** The association between 9 CRLs and PD1, PDL1. *p < 0.05, **p < 0.01, ***p < 0.001.

**Figure S6. TMB analyses, TIDE and drug sensitivity between high- and low-risk groups. (A).** The TMB scores in the high- and low-risk groups. **(B).** The TIDE scores in the high- and low-risk groups. **(C-F).** The correlation between the four types of drugs (bryostatin 1 **(C)**, gemcitabine **(D)**, midostaurin **(E)**, and rapamycin **(F)**) sensitivity and risk scores.

**Figure S7. The PCA and t-SNE of clusters and risk groups and immune correlation analysis in 2 clusters.** **(A-D).** The PCA and t-SNE of clusters and risk groups. **(E).** Heatmap of immune cells in clusters. **(F).** Different expressions of checkpoints in clusters. **(G).** The CDF curve. **p < 0.01, ***p < 0.001.

**Figure S8 The expression levels of 9 CRLs in gliomas and K-M survival curves of different expression level of 9 CRLs. (A).** Box plots showed the expression levels of 9 CRLs used to establish the prognostic signature in gliomas. **(B).** Kaplan–Meier survival analysis showing that eﬀect of LEF1-AS1 expression level on progression-free interval (PFI). **(C-J).** Kaplan–Meier survival analysis showing that eﬀect of HCG15, AC007950.2, PTPRN2-AS1, TRHDE-AS1, AC021739.2, AC008915.2, ARHGAP42-AS1, and LINC01571 expression level on overall survival (OS). ***p < 0.001.

**Figure S9 The relationship between the level of LEF1-AS1 and prognosis of glioma patients in the CGGA database. (A) and (D).** The Kaplan-Meier curves for survival in the CGGA_325 **(A)** and CGGA_693 **(D)** database. **(B) and (E).** The receiver operating characteristic (ROC) curve analyses of LEF1-AS1 expression in predicting 1-, 3-, and 5-year OS in the CGGA_325 **(B)** and CGGA_693 **(E)** database. **(C) and (F).** The AUC of LEF1-AS1 expression in predicting 10-year OS in the CGGA_325 **(C)** and CGGA_693 **(F)** database.

**Supplementary Table S1: 19 cuproptosis-related genes.**

| NFE2L2 | NLRP3 | ATP7B | ATP7A | SLC31A1 |
| --- | --- | --- | --- | --- |
| FDX1 | LIAS | LIPT1 | LIPT2 | DLD |
| DLAT | PDHA1 | PDHB | MTF1 | GLS |
| CDKN2A | DBT | GCSH | DLST |  |

**Supplementary Table S2: Primer list of PCR.**

| Gene Name | Forward primer | Reverse primer |
| --- | --- | --- |
| GAPDH | TGTGTCCGTCGTGGATCTGA | CCTGCTTCACCACCTTCTTGA |
| LEF1-AS1 | CTACCCATCCTCACTGTCAGTC | GGATGTTCCTGTTTGACCTGAGG |

**Supplementary Table S3: Sequence of the applied plasmid.**

| Gene Name | Sequence |
| --- | --- |
| si-NC | CGAACUCACUGGUCUGACC |
| si-LEF1-AS1#1 | GCTGGTCTGCAAGAGACAATT |
| si-LEF1-AS1#2 | GCTCATTCCCAACGTGCAAAG |
